# Muscle-specific lack of Gfpt1 triggers ER stress to alleviate misfolded protein accumulation

**DOI:** 10.1242/dmm.050768

**Published:** 2024-07-25

**Authors:** Ruchen Zhang, Paniz Farshadyeganeh, Bisei Ohkawara, Kazuki Nakajima, Jun-ichi Takeda, Mikako Ito, Shaochuan Zhang, Yuki Miyasaka, Tamio Ohno, Madoka Mori-Yoshimura, Akio Masuda, Kinji Ohno

**Affiliations:** ^1^ Division of Neurogenetics, Center for Neurological Diseases and Cancer, Nagoya University Graduate School of Medicine, Nagoya 466-8550, Japan; ^2^ Institute for Glyco-core Research (iGCORE), Gifu University, Gifu 501-1193, Japan; ^3^ Division of Experimental Animals, Nagoya University Graduate School of Medicine, Nagoya 466-8550, Japan; ^4^ Department of Neurology, National Center Hospital, National Center of Neurology and Psychiatry, Kodaira 187-8775, Japan; ^5^ Graduate School of Nutritional Sciences, Nagoya University of Arts and Sciences, Nisshin 470-0196, Japan

**Keywords:** Glutamine fructose-6-phosphate transaminase 1 (GFPT1), Congenital myasthenic syndrome (CMS), Neuromuscular junction (NMJ), Hexosamine biosynthesis pathway (HBP), Endoplasmic reticulum (ER) stress, Unfolded protein response (UPR)

## Abstract

Pathogenic variants in *GFPT1*, encoding a key enzyme to synthesize UDP-*N*-acetylglucosamine (UDP-GlcNAc), cause congenital myasthenic syndrome (CMS). We made a knock-in (KI) mouse model carrying a frameshift variant in *Gfpt1* exon 9, simulating that found in a patient with CMS. As *Gfpt1* exon 9 is exclusively expressed in striated muscles, *Gfpt1*-KI mice were deficient for Gfpt1 only in skeletal muscles. In *Gfpt1*-KI mice, (1) UDP-HexNAc, CMP-NeuAc and protein *O*-GlcNAcylation were reduced in skeletal muscles; (2) aged *Gfpt1*-KI mice showed poor exercise performance and abnormal neuromuscular junction structures; and (3) markers of the unfolded protein response (UPR) were elevated in skeletal muscles. Denervation-mediated enhancement of endoplasmic reticulum (ER) stress in *Gfpt1*-KI mice facilitated protein folding, ubiquitin-proteasome degradation and apoptosis, whereas autophagy was not induced and protein aggregates were markedly increased. Lack of autophagy was accounted for by enhanced degradation of FoxO1 by increased Xbp1-s/u proteins. Similarly, in *Gfpt1*-silenced C2C12 myotubes, ER stress exacerbated protein aggregates and activated apoptosis, but autophagy was attenuated. In both skeletal muscles in *Gfpt1*-KI mice and *Gfpt1*-silenced C2C12 myotubes, maladaptive UPR failed to eliminate protein aggregates and provoked apoptosis.

## Teaser

**Summary:** Gfpt1 deficiency only in skeletal muscles triggers the unfolded protein response. Maladaptive unfolded protein response due to suppressed autophagy fails to eliminate protein aggregates and provokes apoptosis in skeletal muscles.

## INTRODUCTION

Congenital myasthenic syndromes (CMSs) are rare and heterogeneous inherited neuromuscular disorders caused by pathogenic variants in molecules expressed at the neuromuscular junction (NMJ) (Ohno et al., 2023). CMS is characterized by fatigable muscle weakness, muscle hypoplasia and minor facial anomalies. The phenotypes and severity of CMS depend on the defective gene, which, however, cannot be readily predicted by clinical phenotypes, laboratory findings or electrophysiological studies. Most of the defective genes are specifically expressed at the NMJ, but genes encoding ubiquitously expressed glycosylation enzymes, including *GFPT1*, *DPAGT1*, *ALG2*, *ALG14* and *GMPPB* (Finsterer, 2019), also cause limb-girdle CMS (LG-CMS), in which proximal limb muscles are predominantly affected (Evangelista et al., 2015). However, the underlying pathomechanisms of defective glycosylation enzymes in LG-CMS have not been fully elucidated.

*GFPT1* encodes glutamine fructose-6-phosphate transaminase 1 (GFPT1), which is the first and rate-limiting enzyme of the hexosamine biosynthesis pathway (HBP) to produce uridine diphosphate (UDP) *N*-acetylglucosamine (UDP-GlcNAc), a substrate for *N*- and *O*-linked glycosylation (Ghosh et al., 1960; Paneque et al., 2023). Alternative splicing of *GFPT1* exon 9 (54 bp) generates a ubiquitously expressed short isoform (*GFPT1-S*) excluding exon 9 and a striated muscle-specific long isoform (*GFPT1-L*) including exon 9. Compared to GFPT1-S, the reaction velocity (V_max_) and Michaelis constant (K_M_) for fructose-6-phosphate and inhibition constant (K_i_) for UDP-GlcNAc of GFPT1-L were 53% (Niimi et al., 2001), 215% (DeHaven et al., 2001) and 20% (DeHaven et al., 2001), respectively. Thus, GFPT1-L has a lower enzymatic activity and a higher feedback inhibition by UDP-GlcNAc compared to GFPT1-S. *GFPT1-L* constitutes 80-90% of total *GFPT1* transcripts in skeletal muscles in humans and rodents (DeHaven et al., 2001; Farshadyeganeh et al., 2023). *GFPT1-L* is also expressed in cardiac muscles at a lower level compared to skeletal muscles. In other tissues, *GFPT1-S* is exclusively expressed and no *GFPT1-L* is observed. Most pathogenic variants in *GFPT1* in patients with LG-CMS patients are outside of exon 9 and are expressed throughout the body (Bauche et al., 2017). In contrast, we reported clinical features of a 38-year-old female with LG-CMS with a homozygous pathogenic variant (NM_001244710.2: c.722dupG) in *GFPT1* exon 9 (Matsumoto et al., 2019). The c.722dupG variant in exon 9 predicts a frameshift leading to a premature stop codon in exon 10, yielding a truncated GFPT1 protein (p.G241GfsX39) in skeletal muscles. Issop et al. (2018) knocked out both *Gfpt1-L* and *-S* specifically in skeletal muscle and showed CMS-like phenotypes in the knockout mice. In contrast, specific loss of *Gfpt1-L* in skeletal muscles has not been reported.

Glycosylation is a post-translational modification process that attaches sugar moieties to the proteins or lipids to expand the diversity of these molecules (Yang et al., 2023). Glycan structures have crucial biochemical and physiological roles in folding, quality control, stability, transport and functions of proteins (Moremen et al., 2012; Varki, 2017). Analysis of deficiencies in glycosylation enzymes in animal models and human diseases have disclosed essential roles of glycosyltransferases in mammals (Freeze et al., 2014; Lowe and Marth, 2003; Varki et al., 2009). Protein glycosylation requires sequential coordinated processes in the endoplasmic reticulum (ER) and the Golgi apparatus, and more than 85% of secretory proteins are glycosylated (Steentoft et al., 2013; Zielinska et al., 2010). Similarly, the majority of nuclear and cytoplasmic proteins are also subjected to dynamic *O*-GlcNAcylation (Hart, 2019). For both extracellular and intracellular proteins, malfunctional proteins without proper glycosylation are degraded through the ubiquitin-proteasome pathway (Chen et al., 2011; Dikic, 2017; Pohl and Dikic, 2019). However, excessive overload to the ubiquitin-proteasome pathway by persistently produced malfunctional proteins causes insoluble protein aggregates due to their aggregation-prone nature (Hipp et al., 2019), which are eventually degraded through the autophagy-lysosome pathway (Chen et al., 2011; Dikic, 2017; Pohl and Dikic, 2019). Persistent generation of malfunctional proteins and subsequent protein aggregates induce ER stress and activate the unfolded protein response (UPR) (Haeri and Knox, 2012; Li and Sun, 2021). Misfolded proteins accumulated in the ER bind to 78-kDa glucose-regulated protein (Grp78, also known as Bip, encoded by *Hspa5*) and consequently dissociate Grp78 from UPR sensors that comprise protein kinase RNA-like ER kinase (PERK), inositol-requiring enzyme 1 α (IRE1α, encoded by *Ire1a*) and activating transcription factor 6 (ATF6) (Hetz, 2012). First, for cell survival, PERK released from Grp78 induces the inhibition of protein synthesis through phosphorylation of the eukaryotic translation initiation factor 2α (eIF2α, encoded by *Eif2a*) (Park et al., 2018). Additionally, IRE1α released from Grp78 induces the expression of ER chaperones to enhance proper protein folding, and also ER-associated protein degradation (ERAD) components to trigger the ubiquitin-proteasome pathway, both by generating spliced X-box-binding protein 1 (Xbp1-s) (Park et al., 2021). Second, when these systems fail to normalize the protein quality, the autophagy pathway is activated through an adaptor protein of the early autophagosome, p62 (encoded by *Sqstm1*), to eliminate worn-out proteins, protein aggregates and damaged organelles. Damaged proteins and organelles are then degraded in late autophagosomes by converting light chain 3 (LC3) I to II (Glick et al., 2010; Parzych and Klionsky, 2014). Third, when a large amount of malfunctional proteins provoke persistent ER stress and UPR, PERK released from Grp78 induces the expression of the C/EBP homologous protein Chop (encoded by *Ddit3*) through the Bcl-2-associated X (Bax) pathway to induce cell apoptosis (Hu et al., 2018; Oyadomari and Mori, 2004). Accumulating evidence suggests that the UPR pathways play pivotal roles in the regulation of skeletal muscle mass in degenerative and other types of muscle disorders, as well as in abnormal catabolic states due to increased insulin resistance (Afroze and Kumar, 2019). However, little is known about the relationship between GFPT1-mediated HBP flux for protein glycosylation, UPR and the autophagy pathway in skeletal muscles in CMS.

Here, we generated a knock-in (KI) mouse model carrying a homozygous frameshifting variant in *Gfpt1* exon 9, which simulated a pathogenic variant found in a patient with CMS. We show that the KI mice reduced *Gfpt1* expression and HBP flux in skeletal muscles and developed CMS-like phenotypes with aging, which was accounted for by the induction of ER stress and maladaptive UPR.

## RESULTS

### c.716dupG in *Gfpt1* exon 9 reduces glucose flux to the HBP in skeletal muscles

We generated a KI mouse model carrying a duplicated G nucleotide at position c.716 (c.716dupG) in *Gfpt1* exon 9 (*Gfpt1*-KI mice) using the CRISPR/Cas9 system (Fig. S1A). c.716dupG predicts a premature stop codon in exon 10 and yields a truncated Gfpt1 (p.G239GfsX28). c.716dupG in mouse *Gfpt1* exon 9 is equivalent to the pathogenic variant c.722dupG in *GFPT1* exon 9 found in a patient with CMS (Matsumoto et al., 2019).

**Fig. 1.**
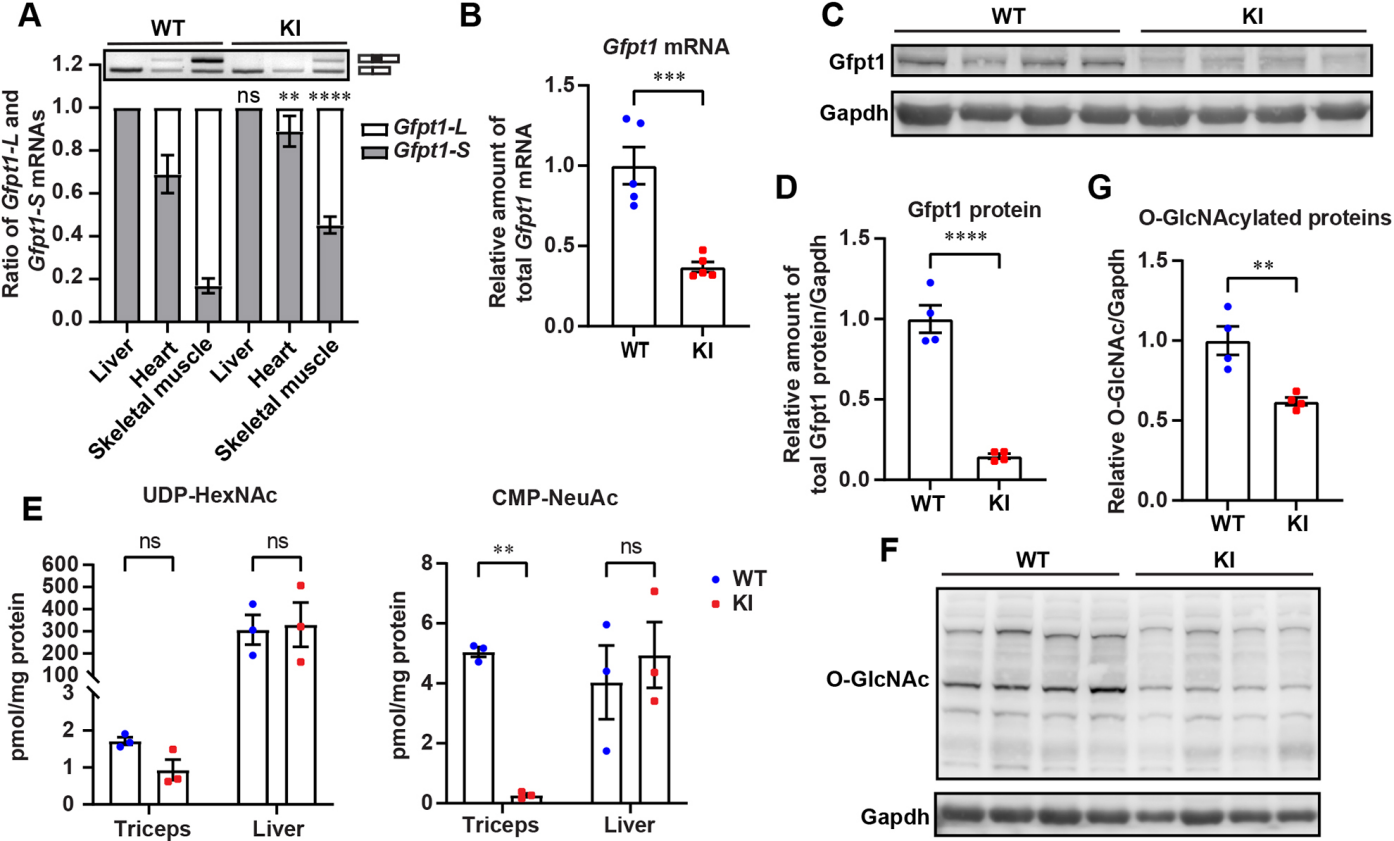
**c.716dupG in *Gfpt1* exon 9 markedly reduced *Gfpt1* mRNA and Gfpt1 protein, as well as HBP flux, in skeletal muscles at 12 months of age.** (A-G) WT and *Gfpt1*-KI mice at 12 months of age were analyzed. (A) Representative RT-PCR and quantification of the ratio of *Gfpt1-L* and *Gfpt1-S* in the indicated tissues (*n*=3 mice each). For statistical tests, samples were compared to the same tissue in WT mice. (B) Quantitative RT-PCR of total *Gfpt1* mRNA in gastrocnemius muscles (*n*=5 mice each). (C,D) Representative immunoblots and quantification of Gfpt1 protein in gastrocnemius muscles (*n*=4 mice each). (E) LC-MS/MS analysis of UDP-HexNAc (UDP-GlcNAc plus UDP-GalNAc) and CMP-NeuAc (*n*=3 mice each). (F,G) Representative immunoblots and quantification of *O*-GlcNAcylated proteins in gastrocnemius muscles (*n*=4 mice each). Mean and s.e.m. are indicated. ns, no significance; **P<0.01; ***P<0.001; ****P<0.0001 (two-way ANOVA followed by Sidak's post hoc test for A,E; two-tailed unpaired Student's *t*-test for B,D,G).

The ratio of *Gfpt1-L* to *Gfpt1-S* was markedly decreased in the cardiac and skeletal muscles in *Gfpt1*-KI mice compared to that in wild-type (WT) mice, suggesting that a *Gfpt1-L* transcript carrying c.716dupG was likely to be degraded by nonsense-mediated mRNA decay (Fig. 1A). In addition, total *Gfpt1* mRNA in the gastrocnemius (GAS) muscles was reduced by ∼65% in *Gfpt1*-KI mice compared to that in WT mice (Fig. 1B). Western blotting also confirmed the reduction of the Gfpt1 protein in GAS muscles by ∼80% in *Gfpt1*-KI mice (Fig. 1C,D). As Gfpt1-L is larger than Gfpt1-S only by 2.0 kDa, Gfpt1-L and Gfpt1-S could not be individually quantified by immunoblotting.

Gfpt1 is the rate-limiting enzyme to generate UDP-GlcNAc in the HBP. To examine whether HBP flux was downregulated by markedly reduced Gfpt1 protein, we quantified UDP-*N*-acetylhexosamine (UDP-HexNAc), which is composed of UDP-GlcNAc and UDP-*N*-acetylgalactosamine (UDP-GalNAc), by liquid chromatography coupled with tandem mass spectrometry (LC-MS/MS). UDP-GlcNAc and UDP-GalNAc are structural isomers with the same molecular mass and could not be differentiated by LC-MS/MS. The total amount of UDP-HexNAc was decreased in the GAS muscles in *Gfpt1*-KI mice, but not in the liver where *Gfpt1-L* was not expressed (Fig. 1E). Moreover, the amount of cytidine monophosphate-*N*-acetylneuraminic acid (CMP-NeuAc), which is produced from UDP-GlcNAc and is the substrate of the sialic acid biosynthetic pathway, was markedly reduced in the skeletal muscles in *Gfpt1*-KI mice (Fig. 1E). As UDP-GlcNAc is a substrate for *O*-GlcNAcylation of proteins (Ghosh et al., 1960; Paneque et al., 2023), we examined *O*-GlcNAcylation levels of proteins. Western blotting showed that the amounts of *O*-GlcNAcylated proteins was reduced by ∼40% in the GAS muscles in *Gfpt1*-KI mice compared to that in WT mice (Fig. 1F,G). These results suggested that loss of Gfpt1-L in skeletal muscles due to c.716dupG markedly reduced the HBP flux and the subsequent *O*-GlcNAcylation of proteins.

RNA sequencing (RNA-seq) analysis of skeletal muscles showed that genes in the glycosylation pathways were either upregulated (*Uap1*, *Dpagt1*, *Alg2* and *Ogt*) or downregulated (*Gnpda1* and *Oga*) in *Gfpt1*-KI mice, which was likely to compensate for lack of Gfpt1 and subsequent suppression of the HBP (Fig. S1E). RNA-seq analysis also showed that genes expressed at the NMJ (*Lrp4*, *Dok7*, *Lama5*, *Lamb2* and *Rapsn*) were upregulated in *Gfpt1*-KI mice, which was likely to compensate for defective NMJ signal transmission (Fig. S1F).

### Muscle-specific deficiency of Gfpt1-L causes CMS-like phenotypes in aged mice

**Fig. 2.**
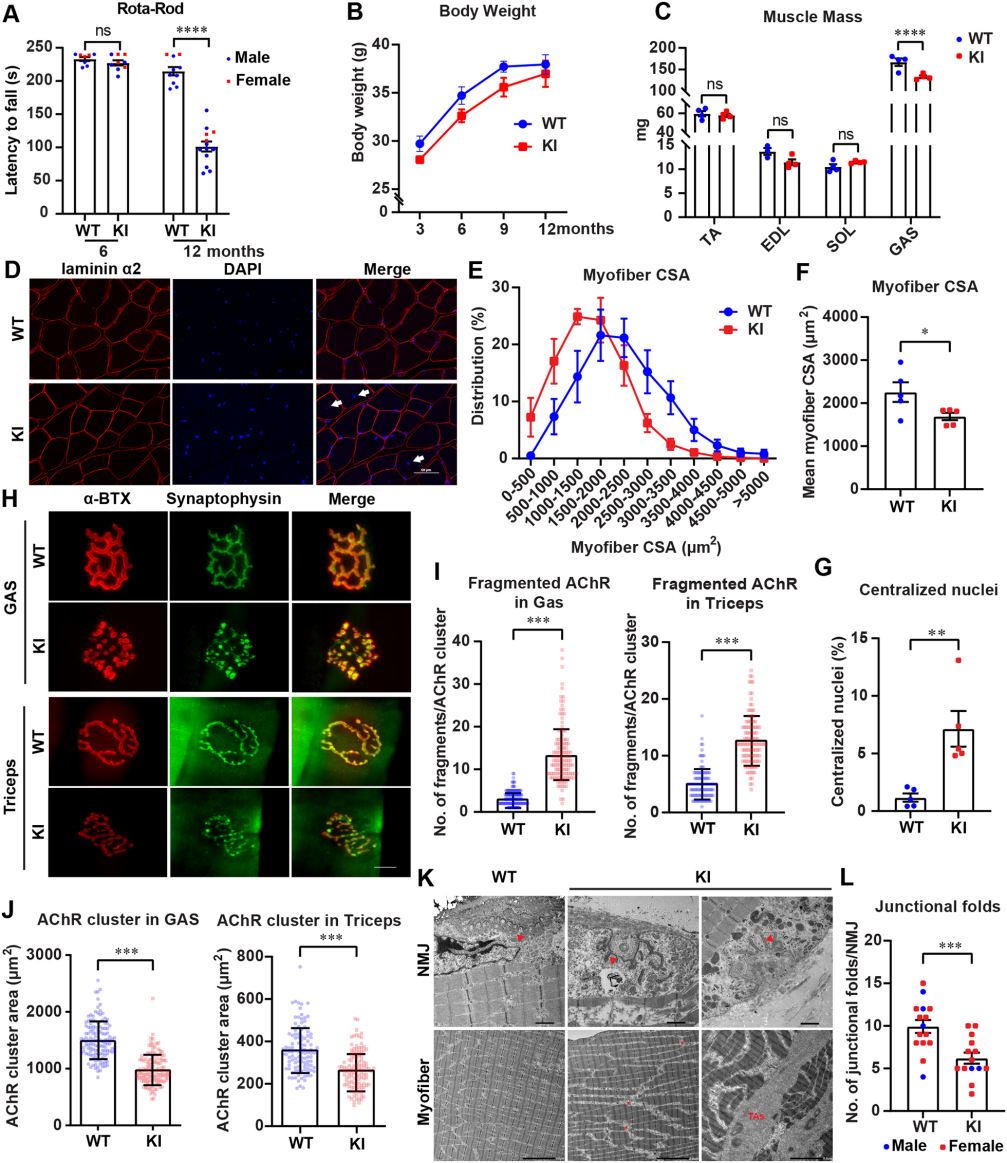
**Muscle-specific Gfpt1 deficiency caused CMS-like phenotypes in *Gfpt1*-KI mice at 12 months of age.** (A) Motor function test using the accelerated rotarod was performed on WT and *Gfpt1*-KI mice at ages 6 and 12 months (*n*=8 to 13 mice each). Males and females are indicated by blue circles and red squares, respectively. (B) Temporal profile of body weights of male WT and *Gfpt1*-KI mice at the indicated ages (*n*=12 mice). Two-way repeated measures ANOVA showed a difference between WT and *Gfpt1*-KI mice (*P*=0.0052), but Sidak's post hoc test showed no difference at either age (*P*=0.26 to 0.90). Body weights of female mice are indicated in Fig. S2A. (C-L) WT and *Gfpt1*-KI mice were analyzed at 12 months of age. (C) Weights of tibialis anterior (TA), extensor digitorum longus (EDL), soleus (SOL) and gastrocnemius (GAS) muscles in WT and *Gfpt1*-KI mice at 12 months of age (*n*=4 mice). Muscle weights of female mice are indicated in Fig. S2B. (D) Representative confocal images of cross-sections of GAS muscles stained with an anti-laminin α2 antibody (red) to label sarcolemma and DAPI (blue) to label nuclei. Arrows point to centralized nuclei. Scale bar: 50 μm. (E,F) Distribution (E) and mean±s.e.m. (F) of cross-sectional areas (CSA) of GAS muscles (*n*≥100 myofibers per image, three images per mouse, five mice each). (G) Percentage of myofibers with centralized nuclei in GAS muscles (*n*≥100 myofibers per image, three images per mouse, five mice each). (H) Representative images of the neuromuscular junctions (NMJs) stained with α-bungarotoxin (α-BTX, red) to label acetylcholine receptor (AChR) and synaptophysin (green) to label the nerve terminal in GAS muscles. Scale bar: 50 μm. (I,J) The ratio of AChR cluster fragments (I) and the AChR cluster areas (J) in the GAS and triceps brachii muscles (*n*=20 to 30 AChR clusters per mouse, five mice each). (K) Representative ultrastructure of the NMJs and myofibers in triceps brachii muscles. Junctional folds (red arrowheads), fat droplets (asterisks), and tubular aggregates (TAs) are indicated. Scale bars: 1 μm (NMJs, top); 5 μm (myofibers, bottom). (L) The number of junctional folds in triceps brachii muscles (*n*=1 to 7 NMJs per mouse, four mice each). Males and females are indicated by blue circles and red squares, respectively. Mean±s.e.m. are indicated. ns, no significance; *P<0.05; **P<0.01; ***P<0.001; ****P<0.0001 (one-way ANOVA followed by Tukey's post hoc test for A; two-way ANOVA followed by Sidak's post hoc test for B,C; two-tailed unpaired *t*-test for F,G,I,J,L). Note that male and female mice were used in A and L, whereas only male mice were used in the other panels and the other figures.

We next examined whether *Gfpt1*-KI mice showed similar phenotypes to those in the patient with *GFPT1*-associated CMS. The rotarod test at 6 months of age in *Gfpt1*-KI mice showed no sign of muscle weakness (Fig. 2A). However, at 12 months of age, *Gfpt1*-KI mice stayed on the rotarod for a shorter duration (by ∼55%) than that for WT mice (Fig. 2A). Thus, *Gfpt1*-KI mice developed muscle weakness at 12 months of age but not at 6 months of age.

As the patient with *GFPT1*-CMS carrying the homozygous c.722dupG variant showed diffuse muscle atrophies by computer tomography (CT) scanning (Matsumoto et al., 2019), we examined whether *Gfpt1*-KI mice developed similar phenotypes. Body weights of *Gfpt1*-KI mice were smaller than those of WT mice over the course of aging (Fig. 2B; Fig. S2A). The weights of the tibialis anterior, extensor digitorum longus and soleus muscles were not different between WT and *Gfpt1*-KI mice at 12 months of age, whereas the weight of the GAS muscle was lower in *Gfpt1*-KI mice than in WT mice (Fig. 2C; Fig. S2B). Similarly, truncal CT images at 12 months of age showed that the cross-sectional area of visceral fat was increased and that of the skeletal muscles was decreased in *Gfpt1*-KI mice (Fig. S2C,D). We then stained the GAS muscles using an antibody against laminin α2 (encoded by *Lama2*) and DAPI (Fig. 2D). Cross-sectional areas of myofibers in GAS muscles of *Gfpt1*-KI mice at 12 months of age were smaller than those in age-matched WT mice (Fig. 2E,F). In addition, the majority of WT myofibers showed peripherally located nuclei, whereas *Gfpt1*-KI myofibers occasionally exhibited centralized nuclei, suggesting myofiber regeneration in GAS muscles in *Gfpt1*-KI mice (Fig. 2G). Thus, 12-month-old *Gfpt1*-KI mice exhibited atrophy and regeneration of the GAS muscle. In contrast, quantitative real-time (qRT-PCR) of *Myh7* (expressed in type I myofibers), *Myh2* (expressed in type IIA myofibers) and *Myh4* (expressed in type IIb myofibers) in the soleus muscles at 12 months of age showed that *Myh4* expression tended to be low in *Gfpt1*-KI mice, although no statistical significance was observed (Fig. S4A).

We next examined the morphology of the NMJ. Staining of acetylcholine receptor (AChR) clusters and synaptophysin at the nerve terminal in the GAS and triceps brachii muscles in WT and *Gfpt1*-KI mice at 12 months of age showed that, in contrast to the pretzel-like structure of AChR clusters in WT myofibers, *Gfpt1*-KI myofibers showed fragmentation of AChR clusters (Figs 2H,I; Fig. S4F). Similarly, the areas of AChR clusters were also reduced in *Gfpt1*-KI myofibers compared to those in WT myofibers (Fig. 2J). Ultrastructural analysis of the triceps brachii muscles in *Gfpt1*-KI mice at 12 months of age showed abnormal fat droplets in the muscle intermedium and decreased numbers of junctional folds at the NMJ compared to those in WT mice (Fig. 2K,L). Moreover, *Gfpt1*-KI myofibers showed tubular aggregates (Fig. 2K), which was frequently observed in patients with *GFPT1*-CMS (Selcen et al., 2013). In contrast to the abnormal AChR clusters at 12 months of age in GAS myofibers in *Gfpt1*-KI mice, AChR clusters were minimally fragmented at 6 months of age in *Gfpt1*-KI mice (Fig. S4B,C). Taken together, *Gfpt1* c.716dupG compromised motor functions, damaged myofibers and affected AChR clusters and NMJ ultrastructures in aged mice.

### *Gfpt1*-KI provokes ER stress and activates Grp78-mediated UPR in skeletal muscles

**Fig. 3.**
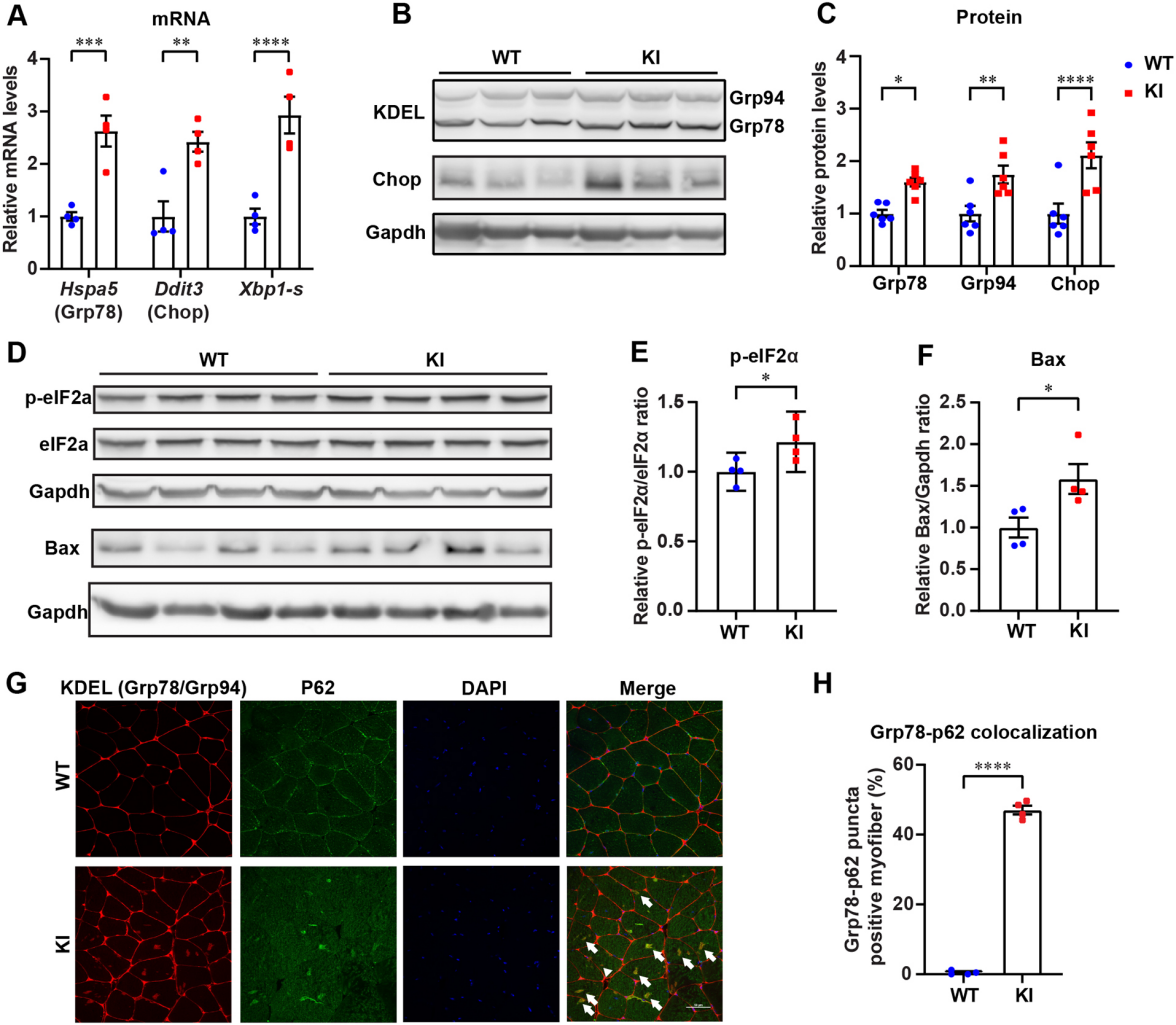
***Gfpt1*-KI activated the UPR, which subsequently promoted translational suppression, protein folding, early autophagy and apoptosis in skeletal muscles.** (A) Quantitative RT-PCR of *Hspa5*, *Ddit3* and *Xbp1-s* in the GAS muscles in WT and *Gfpt1*-KI mice (*n*=4 mice each). (B,C) Representative immunoblots and quantification of Grp78, Grp94 and Chop in the GAS muscles in WT and *Gfpt1*-KI mice (*n*=6 mice each). (D-F) Representative immunoblots and quantification of p-eIF2α, eIF2α and Bax in the GAS muscles in WT and *Gfpt1*-KI mice (*n*=4 mice each). (G) Representative confocal images of cross-sections of myofibers stained with antibodies against KDEL (Grp78/Grp94) and p62 in the GAS muscles in WT and *Gfpt1*-KI mice. Arrows indicate colocalized Grp78-p62. Scale bar: 50 μm. (H) Percentage of the myofibers with KDEL (Grp78)-p62 colocalized puncta in WT and *Gfpt1*-KI GAS muscles (*n*=4 mice each). (A,C,E,F,H) Mean and s.e.m. are indicated. *P<0.05; **P<0.01; ***P<0.001; ****P<0.0001 (two-tailed unpaired Student's *t*-test).

Previously reported muscle-specific knockout of both *Gfpt1-L* and *Gfpt1-S* showed the proliferation of sarcoplasmic reticulum and Golgi apparatus (Issop et al., 2018), which is often observed under ER stress (Brauers et al., 2017). We showed above that muscle-specific lack of Gfpt1-L in *Gfpt1*-KI mice markedly reduced UDP-GlcNAc, CMP-NeuAc and protein *O*-GlcNAcyaltion (Fig. 1). Hypoglycosylated and misfolded proteins due to Gfpt1-L deficiency in skeletal muscles were likely to cause ER stress and activate the UPR by binding the chaperone protein Grp78. As stated in the Introduction, this process facilitates proteostasis and autophagy for cell survival, or apoptosis depending on the duration and degree of ER stress (Griesemer et al., 2014; Ren et al., 2021; Wang et al., 2009). In the autophagy pathway, Grp78 binds to misfolded proteins and recruits p62 in the cytoplasm, which subsequently delivers the misfolded proteins to the autophagosomes for lysosomal degradation (Cha-Molstad et al., 2016). We next examined whether ER stress-induced UPR was activated in skeletal muscles in *Gfpt1*-KI mice at 12 months of age. Expression levels of the UPR-induced genes *Hspa5* (encoding Grp78), *Ddit3* (encoding Chop) and *Xbp1* (encoding the splice isoform Xbp1-s) were markedly increased in skeletal muscles in *Gfpt1*-KI mice (Fig. 3A). Similarly, protein expression levels of the Grp78 and Grp94 (encoded by *Lpg3*) chaperones, as well as Chop, were upregulated (Fig. 3B,C). In addition, phosphorylation of eIF2α was elevated in skeletal muscles in *Gfpt1*-KI mice, which suppresses translation for cell survival (Fig. 3D,E). Furthermore, Bax expression was induced in skeletal muscles in *Gfpt1*-KI mice (Fig. 3D,F), indicating the induction of apoptosis. We found that more than 40% of *Gfpt1-*KI myofibers exhibited Grp78 and p62 colocalization in the cytoplasm, which was much higher than that in WT myofibers (Fig. 3G,H). Taken together, Grp78-mediated UPR was activated in skeletal muscles in *Gfpt1*-KI mice at 12 months of age. However, protein aggregates were not efficiently eliminated.

In contrast to the abnormal colocalization of Grp78 and p62 at 12 months of age, *Gfpt1*-KI mice showed no colocalization of Grp78 and p62 at 6 months of age (Fig. S4E). Instead, we observed that the expression of heat shock protein 70 (Hsp70), which is a key molecule to maintain proteostasis (Alagar Boopathy et al., 2022), was markedly increased at 6 months of age in *Gfpt1*-KI mice (Fig. S4F,G). Thus, reduced protein glycosylation due to Gfpt1-L deficiency was likely to be successfully compensated for at 6 months of age in *Gfpt1*-KI mice.

### Denervation of the sciatic nerve in *Gfpt1*-KI mice exacerbates ER stress-induced UPR and protein aggregates in skeletal muscles

**Fig. 4.**
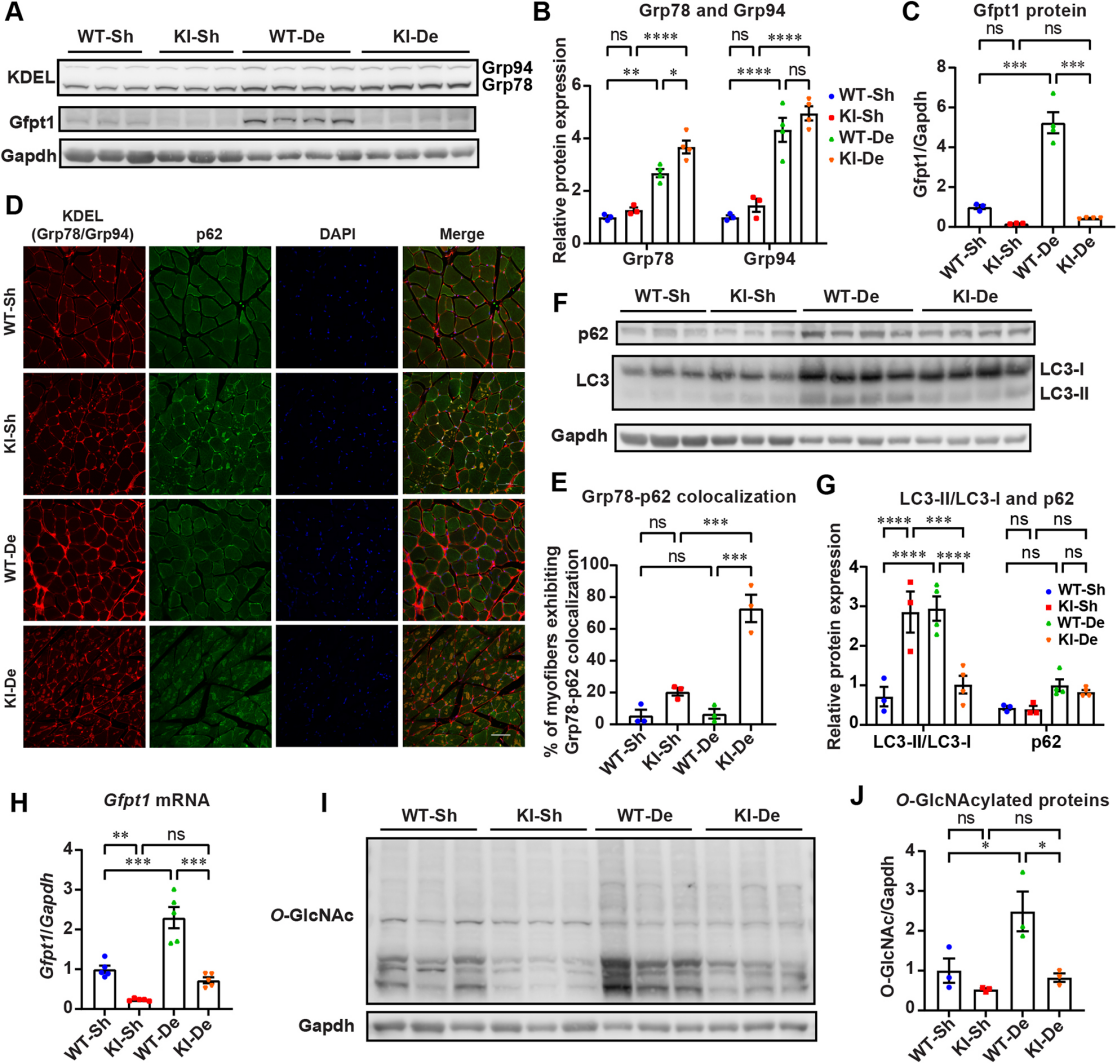
**Denervation failed to activate the hexosamine biosynthesis pathway, markedly enhanced protein folding and compromised autophagy-mediated protein degradation in *Gfpt1*-KI mice.** (A-C) Representative immunoblots and quantification of Grp78, Grp94 and Gfpt1 in the GAS muscles in WT and *Gfpt1*-KI mice (*n*=3 to 4 mice each). Sh, sham-operated; De, denervated. (D) Representative confocal images of cross-sections of myofibers stained with antibodies against KDEL (Grp78/Gpr94) and p62 in the GAS muscles in WT and *Gfpt1*-KI mice. Scale bar: 50 μm. (E) Quantification of D showing the percentage of myofibers with Grp78-p62 colocalization in the GAS muscles in WT and *Gfpt1*-KI mice (*n*=3 mice each). (F,G) Representative immunoblots and quantification of LC3 and p62 in the GAS muscles in WT and *Gfpt1*-KI mice (*n*=4 mice each). (H) Quantitative RT-PCR of total *Gfpt1* in GAS muscles in WT and *Gfpt1*-KI mice (*n*=5 mice each). (I,J) Representative immunoblots and quantification of *O*-GlcNAcylated proteins in GAS muscles in WT and *Gfpt1*-KI mice (*n*=3 mice each). (B,C,E,G,H,J) Mean and s.e.m. are indicated. ns, no significance; *P<0.05; **P<0.01; ***P<0.001; ****P<0.0001 (one-way ANOVA followed by Tukey's post hoc test).

*Gfpt1*-KI mice showed muscle weakness at 12 months of age but not at 6 months (Fig. 2A), suggesting that persistent ER stress might have deteriorated muscle force. Previous reports showed that denervation enhanced ER stress and subsequently activated autophagy (O'Leary and Hood, 2009; Yang et al., 2021). In addition, in *Gfpt1-L/S* knockout mice, glypican-1 (encoded by *Gpc1*), a marker for denervation, was markedly increased in skeletal muscle (Issop et al., 2018). In *Gfpt1*-KI mice, RNA-seq of skeletal muscles similarly showed that expression of *Gpc1* was increased 1.40-fold and that expression of *Chrng* and *Scn5a*, other markers of denervation, was also increased 1.28- and 1.28-fold, respectively. Although mild functional denervation was likely to be already present in *Gfpt1*-KI mice, we physically cut the right sciatic nerve to enhance the ER stress in *Gfpt1*-KI mice at 12 months of age. The denervated GAS muscles were analyzed 4 weeks later. In both WT and *Gfpt1*-KI muscles, denervation increased the expression of Grp78 and Grp94, indicating the induction of ER stress (Fig. 4A,B). Similarly, in both WT and *Gfpt1*-KI muscles, denervation increased the expression of the muscle-specific E3 ubiquitin ligase MuRF1 (encoded by *Trim63*), indicating the activation of the ubiquitin-proteasome pathway (Fig. S3A,B). When autophagy is activated, LC3-I is cleaved by a cysteine protease, Atg4, and LC3-II is generated (Tanida et al., 2004). In the GAS muscles in WT mice, denervation increased the LC3-II/LC3-I ratio but did not increase the Grp78-p62 colocalization (Fig. 4D-G), indicating that autophagy efficiently removed misfolded/unfolded proteins. In contrast, in the GAS muscles in *Gfpt1*-KI mice, denervation decreased the LC3-II/LC3-I ratio and increased the Grp78-p62 colocalization (Fig. 4D-G), indicating that autophagy was suppressed and that misfolded/unfolded proteins were accumulated. In addition, in both WT and *Gfpt1*-KI muscles, denervation increased the expression of Chop and Bax (Fig. S3A,C,D), indicating that apoptosis was induced. However, there was no statistical difference in the expression of Chop and Bax between WT and *Gfpt1*-KI muscles. Taken together, in both WT and *Gfpt1*-KI muscles, denervation activated protein folding, ubiquitin-proteasome degradation and apoptosis. In contrast, autophagy was efficiently induced only in WT muscles but not in *Gfpt1*-KI muscles.

### Gfpt1 is a UPR mediator and lack of Gfpt1 compromises the UPR, which culminates in attenuated autophagy and enhanced apoptosis

Xbp1-s induced by ER stress directly increases Gfpt1 expression, and the subsequent increase of UDP-GlcNAc helps proper glycosylation to prevent the generation of misfolded/unfolded proteins and alleviates ER stress (Wang et al., 2014). Thus, Gfpt1 is one of key molecules to mitigate ER stress. Indeed, we found that denervation increased the expression of *Gfpt1* mRNA and Gfpt1 protein in the GAS muscles in WT mice (Fig. 4A,C,H). Similarly, denervation tended to increase the expression of *Gfpt1* mRNA and Gfpt1 protein in the GAS muscles in *Gfpt1*-KI mice, but the increases were markedly lower compared to those in WT mice (Fig. 4A,C,H). In addition, denervation increased *O*-GlcNAcylation levels in the GAS muscles in WT mice but not in *Gfpt1*-KI mice (Fig. 4I,J).

**Fig. 5.**
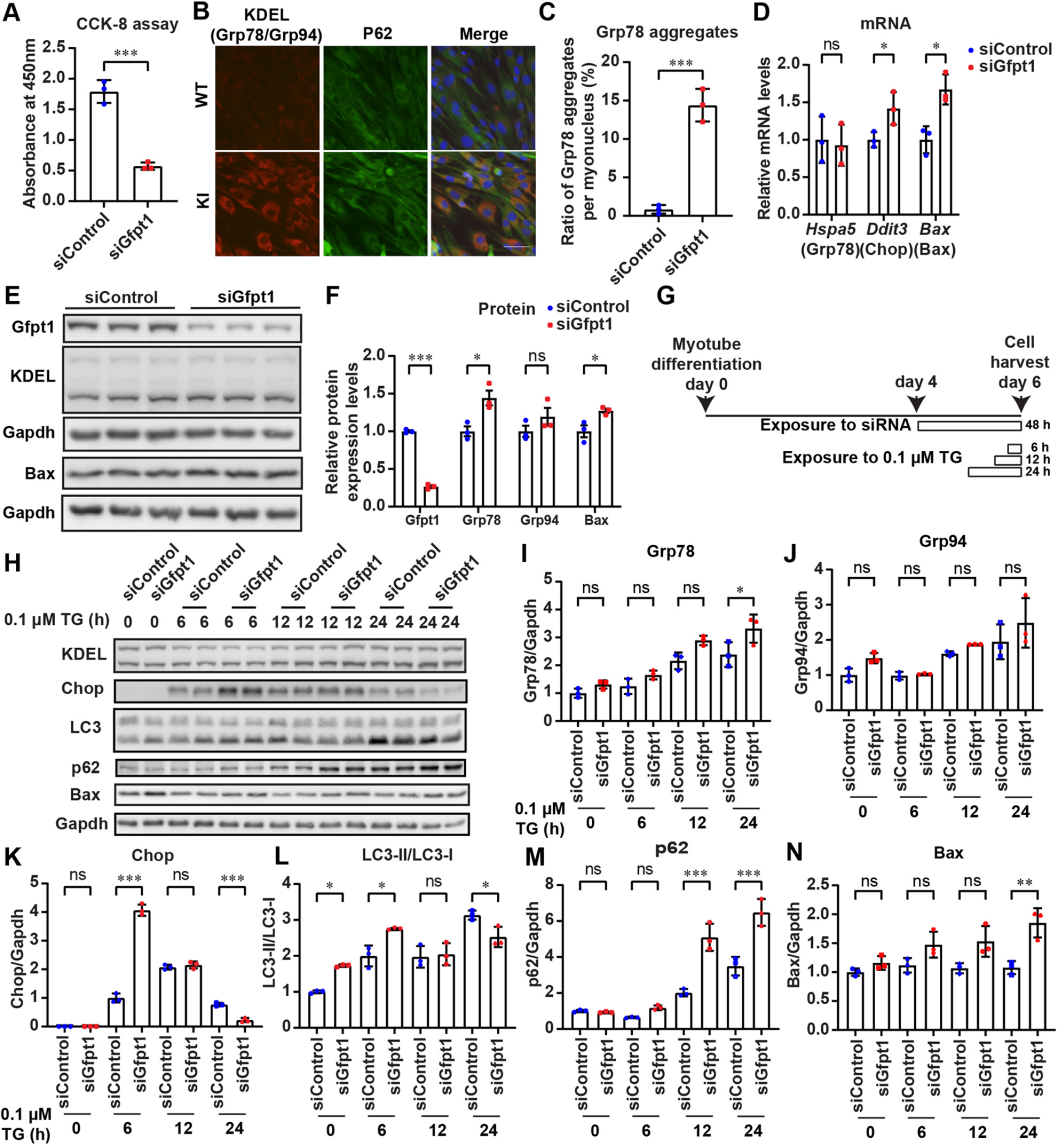
**Thapsigargin induced the UPR in C2C12 myotubes, and Gfpt1 deficiency switched the cell fate from the activation of autophagy to the induction of apoptosis.** (A) Estimation of cell viability by CCK-8 of C2C12 myoblasts transfected with scrambled siRNA (siControl) or siGfpt1 for 48 h (*n*=3 dishes each). (B) Representative images of C2C12 myotubes stained with antibodies against KDEL (Grp78/Grp94) and p62. Scale bar: 50 μm. (C) Quantification of B showing the ratio of Grp78-positive aggregates per myonucleus in C2C12 myotubes transfected with siControl or siGfpt1 (*n*=3 dishes each). (D) Quantitative RT-PCR of *Hspa5*, *Ddit3* and *Bax* in C2C12 myotubes transfected with siControl or siGfpt1 (*n*=3 dishes each). (E,F) Representative immunoblots and quantification of Gfpt1, Grp78, Grp94 and Bax in myotubes transfected with siControl or siGfpt1 (*n*=3 dishes each). (G) Temporal profile of thapsigargin (TG)-mediated induction of UPR in C2C12 myotubes for H-N. Note that siRNA was added 4 days before harvesting cells for B-F. (H-N) Representative immunoblots and quantification of the indicated proteins and the LC3-II/LC3-I ratio in C2C12 myotubes transfected with siControl or siGfpt1 (*n*=3 dishes each). (A,C,D,F,I-N) Mean and s.d. are indicated. ns, no significance; *P<0.05; **P<0.01; ***P<0.001 (two-tailed unpaired Student's *t*-test for A,C; two-way ANOVA followed by Sidak's post hoc test for D,F,I-N).

To investigate the effects of *Gfpt1* knockdown on the UPR in myogenic cells, we transfected C2C12 myoblasts with an siRNA against *Gfpt1* (siGfpt1) (Fig. 5E) and induced myotube differentiation. We found that inhibition of *Gfpt1* (1) reduced cell viability (Fig. 5A); (2) accumulated Grp78-positive signals around nuclei without p62 colocalization (Fig. 5B,C); (3) increased the expression of the Grp78 protein to enhance protein folding (Fig. 5E,F); and (4) increased the expression of *Ddit3* and *Bax* mRNA, as well as the Bax protein, to induce apoptosis (Fig. 5D-F). These results were similar to those observed in the GAS muscles in *Gfpt1*-KI mice except for the lack of cytoplasmic Grp78-p62 colocalization. Thus, Gfpt1 deficiency in C2C12 myotubes induced the apoptosis pathway and compromised cell survival.

We next examined the effects of Gfpt1 deficiency under ER stress in C2C12 myotubes. *Gfpt1*-silenced C2C12 myotubes were treated with 0.1 μM thapsigargin (TG), an ER stress inducer, for 6, 12 and 24 h (Fig. 5G). First, in siControl- and siGfpt1-treated C2C12 myotubes, TG increased the expression of the Grp78 chaperone as well as the Grp94 chaperone to similar levels, indicating that *Gfpt1* silencing had no effect on the TG-induced enhancement of protein folding (Fig. 5H-J). Second, in siControl-treated C2C12 myotubes, TG increased the expression of Chop (Fig. 5H,K), and *Gfpt1* silencing accelerated and enhanced the expression of Chop. Third, in siControl-treated C2C12 myotubes, TG gradually increased the LC3-II/LC3-I ratios, suggesting the induction of autophagy (Fig. 5H,L). *Gfpt1* silencing accelerated the increase of the LC3-II/LC3-I ratios at the baseline at 6 h, but suppressed the increase at 12 h. In addition, *Gfpt1* silencing enhanced the gradual increase of p62 (Fig. 5H,M). The suppressed LC3-II/LC3-I ratio and the enhanced p62 expression at 24 h in siGfpt1-treated C2C12 myotubes indicate that *Gfpt1* silencing attenuated TG-induced autophagy and exacerbated protein aggregates. Instead, the apoptosis marker Bax was induced in siGfpt1-treated, but not in siControl-treated, C2C12 myotubes (Fig. 5H,N). Taken together, *Gfpt1* silencing attenuated the effects of Gfpt1 to enhance the glycosylation of misfolded/unfolded proteins, which consequently changed the cell fate from the activation of autophagy to the induction of cell apoptosis.

### Lack of Gfpt1 in the denervated skeletal muscles compromises the roles of FoxO1 in inducing autophagy

**Fig. 6.**
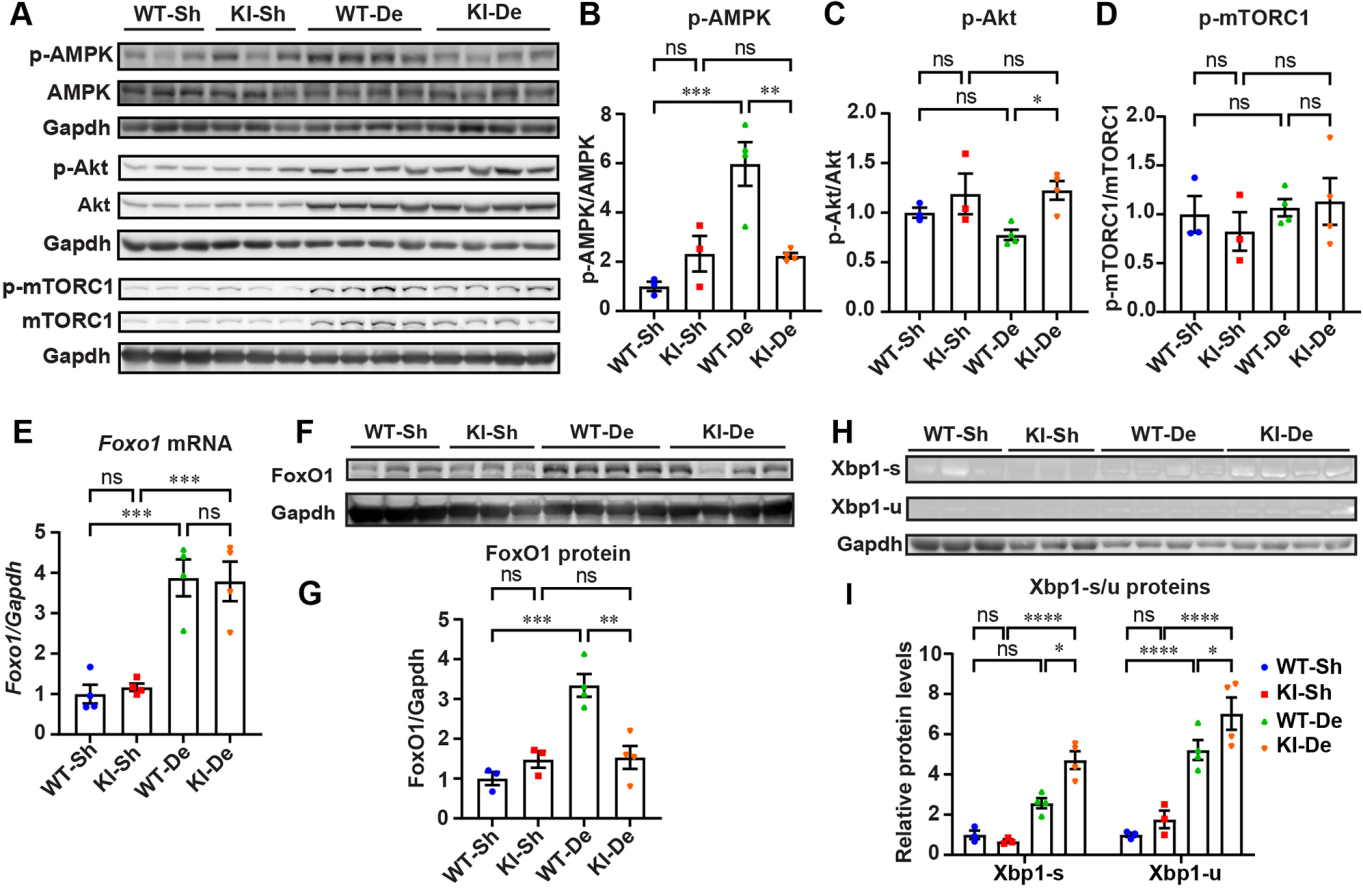
**Denervation had no effect on p-mTOR, but increased FoxO1 in WT mice, which, however, was compromised in *Gfpt1*-KI mice.** (A) Representative immunoblotting of molecules in the AMPK/mTORC1 and Akt/mTORC1 pathways in the GAS muscles in WT and *Gfpt1*-KI mice. Sh, sham-operated; De, denervated. (B-D) Quantification of p-AMPK/AMPK (B), p-Akt/Akt (C) and p-mTOR/mTOR (D) (*n*=3 mice each). (E) Quantitative RT-PCR of *Foxo1* in GAS muscles in WT and *Gfpt1*-KI mice (*n*=4 mice each). (F,G) Representative immunoblotting and quantification of FoxO1 in GAS muscles in WT and *Gfpt1*-KI mice (*n*=3 to 4 mice each). (H,I) Representative immunoblotting and quantification of Xbp1-s and Xbp1-u in the GAS muscles in WT and *Gfpt1*-KI mice (*n*=4 mice each). (B-E,G,I) Mean and s.e.m. are indicated. ns, no significance; *P<0.05; **P<0.01; ***P<0.001; ****P<0.0001 (one-way ANOVA followed by Tukey's post hoc test).

The Akt/mTORC1 and AMPK/mTORC1 pathways ubiquitously regulate autophagy (Alers et al., 2012; Heras-Sandoval et al., 2014; Rashid et al., 2015). We found that denervation decreased phosphorylation of Akt (p-Akt) and increased phosphorylation of AMPK (p-AMPK) in skeletal muscles in WT mice, but not in *Gfpt1*-KI mice (Fig. 6A-C). Both decreased p-Akt and increased p-AMPK lead to the inhibition of autophagy by downregulating the phosphorylation of mTORC1 (p-mTORC1) (Alers et al., 2012; Heras-Sandoval et al., 2014; Rashid et al., 2015). However, denervation did not change p-mTORC1 in either WT or *Gfpt1*-KI mice (Fig. 6A,D), suggesting that the attenuation of autophagy in the denervated skeletal muscle in *Gfpt1*-KI mice was unlikely to be mediated by the mTOR signaling pathway. We next examined the expression of forkhead box O1 (FoxO1), which triggers the expression of autophagy-related genes in the nucleus and also activates autophagy by associating with autophagy proteins (e.g. Atg7) in the cytoplasm (Cheng, 2019). As previously reported (Fjallstrom et al., 2014), denervation markedly increased the expression of *Foxo1* mRNA and FoxO1 protein in the GAS muscles in WT mice (Fig. 6E-G). In *Gfpt1*-KI mice, denervation similarly increased the expression of *Foxo1* mRNA, but the increase of FoxO1 protein was much less than that in denervated WT GAS muscles. Xbp1-s and Xbp1-u bind to FoxO1 to facilitate its proteasomal degradation (Zhao et al., 2013; Zhou et al., 2011). We found that the expression of Xbp1-s and Xbp1-u proteins was increased in denervated GAS muscles in *Gfpt1*-KI mice compared to that in WT mice (Fig. 6H,I). Thus, in the denervated GAS muscles in *Gfpt1*-KI mice, the induction of Xbp1-s and Xbp1-u was likely to have degraded FoxO1, which subsequently attenuated autophagy.

## DISCUSSION

Loss-of-function variants in *GFPT1* cause LG-CMS. We previously reported a homozygous NM_001244710.2: c.722dupG variant in *GFPT1* exon 9 in a 38-year-old female with LG-CMS (Matsumoto et al., 2019). Human *GFPT1* and mouse *Gfpt1* are 90.6% identical at the cDNA level and 99.1% identical at the amino acid level. In this study, we generated a mouse model with a frameshift mutation, c.716dupG (Fig. S1A), which was equivalent to the patient variant, c.722dupG. *RAPSN* is the only gene associated with CMS for which pathogenic variants found in patients have been analyzed in KI mice, to the best of our knowledge (Xing et al., 2019; 2021). Compared to muscle-specific knockout of both *Gfpt1-L* and *Gfpt1-S* in a previous report (Issop et al., 2018), our mice were deficient for only *Gfpt1-L*. *GFPT1* exon 9 generating *GFPT1-L* is exclusively expressed in striated muscles in both humans and mice (Farshadyeganeh et al., 2023). Thus, the patient was predicted to express the mutant GFPT1-L only in striated muscles, and GFPT1-S expression in the other tissues should remain intact. Our patient, as well as previously reported patients with *GFPT1*-CMS (Ohno et al., 2023), lacked overt cardiac phenotypes. Similarly, the total amount of Gfpt1 protein was preserved in the heart in *Gfpt1*-KI mice (Fig. S1C,D). We thus restricted our analysis to skeletal muscles. We observed that c.716dupG markedly reduced *Gfpt1-L* mRNA in skeletal muscles (Fig. 1A,B), which was likely to be accounted for by nonsense-mediated mRNA decay. Similarly, the total amount of Gfpt1 protein was markedly reduced in skeletal muscles in *Gfpt1*-KI mice (Fig. 1D,E). At 6 months of age, *Gfpt1*-KI mice did not show muscle weakness (Fig. 2A) and exhibited normal AChR clusters with only occasional fragmentation (Fig. S4B,C). However, at 12 months of age, *Gfpt1*-KI mice showed muscle weakness/fatigue (Fig. 2A), atrophic myofibers (Fig. 2D-F), compromised AChR clusters (Fig. 2H-J), simplified NMJ ultrastructures (Fig. 2K,L) and tubular aggregates (Fig. 2K,L). Tubular aggregates are observed in skeletal muscles in patients with *GFPT1*-CMS (Selcen et al., 2013) and in mice with muscle-specific *Gfpt1* knockout (Issop et al., 2018). Thus, *Gfpt1*-KI mice showed late-onset symptoms that were similar to those in patients with *GFPT1*-CMS.

**Fig. 7.**
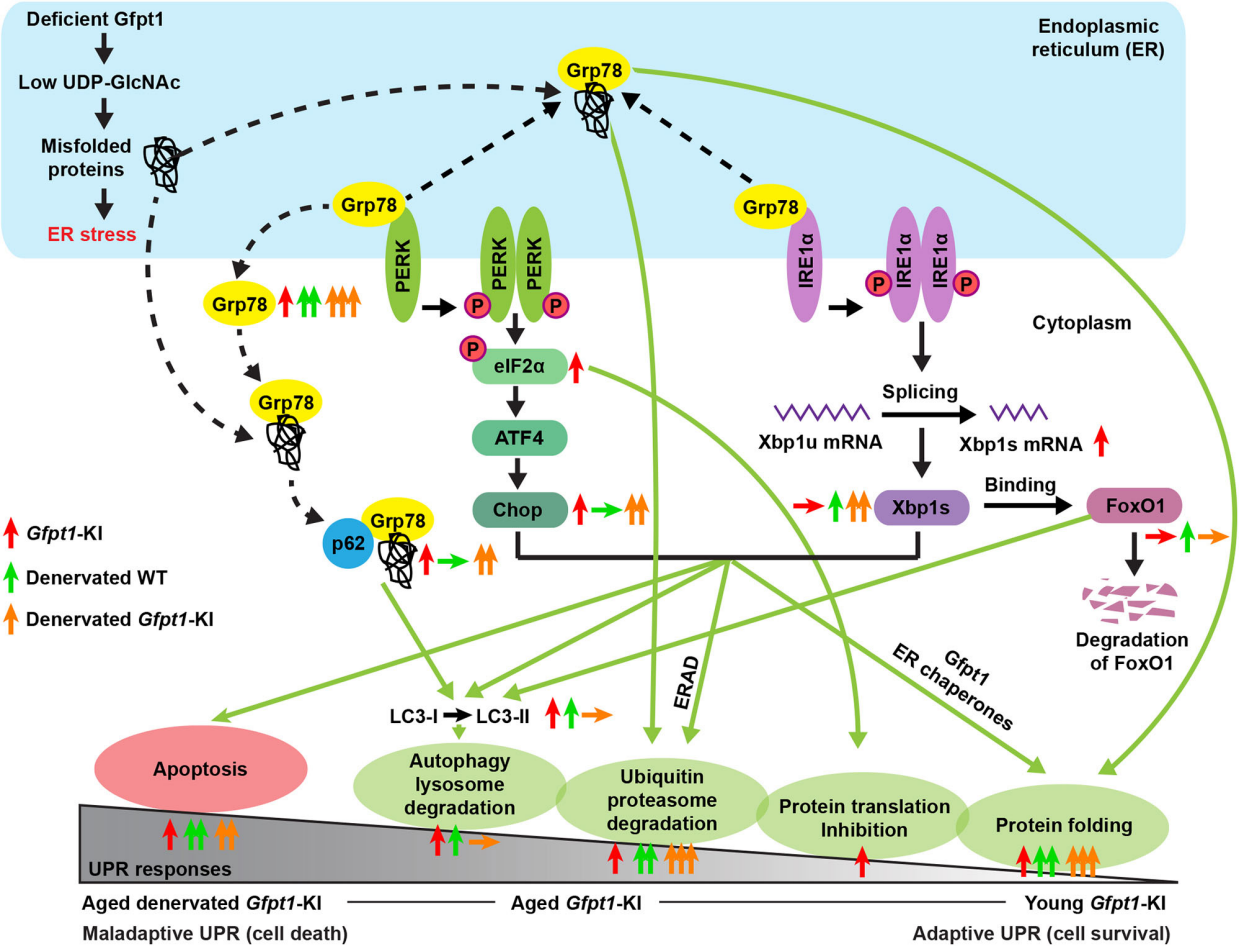
**Schematic summary of the UPR.** The molecules addressed in this study are schematically shown. Up and down arrows indicate upregulation and downregulation of molecules and UPR events in *Gfpt1*-KI mice compared to those in WT mice, respectively. The numbers of arrows indicate the degrees of changes.

Proper glycosylation of proteins is essential for correct protein folding (Jayaprakash and Surolia, 2017). GFPT1 is the first rate-limiting enzyme of HBP that generates UDP-GlcNAc for *N*-linked and *O*-linked glycosylation (Ghosh et al., 1960; Paneque et al., 2023). Activation of GFPT1 by introducing the G451E gain-of-function mutation suppresses the formation of aggregates made by metastable and insoluble proteins in mammalian cells by activating the HBP (Horn et al., 2020). Thus, we hypothesized that skeletal muscles in *Gfpt1*-KI mice might be subjected to ER stress. We indeed found that, in *Gfpt1*-KI muscles at 12 months of age, Grp78-p62 colocalization was increased, suggesting the formation of protein aggregates (Fig. 3G,H). The induced UPR suppressed protein translation (p-eIF2α), enhanced chaperone expression (Grp78, Grp94), induced early autophagy (*Xbp1-s* and p62) and activated apoptosis (Chop and Bax) (Fig. 3). A previous study showed that muscle-specific lack of *Gfpt1-L* and *Gfpt1-S* caused abnormal expansion of the ER in myofibers by electron microscopy (Issop et al., 2018), which were likely to have represented overloaded ER stress. However, Grp78-p62 colocalization was not found in *Gfpt1*-KI muscles at 6 months of age (Fig. S4E), indicating that deficient Gfpt1 initially caused adaptive UPR that inhibited aggregation of misfolded proteins and triggered cell survival. These results suggested that lack of Gfpt1 caused misfolded/unfolded proteins and triggered maladaptive UPR in skeletal muscles with aging (Fig. 7).

Patients with CMS often exhibit muscle atrophy and/or hypotrophy, likely due to the compromised NMJ signal transmission (Ohno et al., 2023). Similar muscle atrophy/hypotrophy is also observed in mouse models of CMS (Webster, 2018). Conversely, defects in protein homeostasis in the ER and malfunctioning autophagy affect the morphology and functions of the NMJ. Du et al. (2016) showed that, in skeletal muscles, chemically induced ER stress accelerated AChR degradation by promoting endocytosis. Similarly, Carnio et al. (2014) showed that muscle-specific autophagy inhibition by Atg7 knockout caused fragmented AChR clusters and NMJ instability. We showed that ER stress followed by the UPR was induced in skeletal muscles in *Gfpt1*-KI mice (Fig. 3). In addition, in both WT and *Gfpt1*-KI mice, the disruption of NMJ signal transmission by denervation accelerated the UPR, which subsequently activated protein folding (Fig. 4A,B), ubiquitin-proteasome degradation (Fig. S3A,B) and apoptosis (Fig. S3A,C,D). In contrast, denervation efficiently induced autophagy to eliminate protein aggregates in WT mice but not in *Gfpt1*-KI mice (Fig. 4D-G). Similarly, in C2C12 myotubes, the induction of ER stress by TG activated autophagy, but *Gfpt1* silencing attenuated the activation of autophagy (Fig. 5G-J,L,M). In neurodegenerative diseases, ER stress failed to induce autophagy, which subsequently exacerbates protein aggregates (Rashid et al., 2015). For example, in transgenic mice carrying mutant Huntingtin (mHtt), simulating Huntington's disease, or mutant superoxide dismutase 1 (mSOD1), simulating familial amyotrophic lateral sclerosis (fALS), knockdown of *Xbp1* that enhances the protein folding and proteasome pathways rather improved neuronal survival and motor performance, and drastically enhanced the clearance of protein aggregates by promoting autophagy (Hetz et al., 2009; Vidal et al., 2012). This indicates that misfolded/unfolded mHtt and mSOD1 could be efficiently eliminated only by autophagy, but this process was suppressed by the presence of Xbp1-s (Rashid et al., 2015). Thus, the suppression of autophagy even in the presence of overloaded misfolded/unfolded proteins in the denervated GAS muscles in *Gfpt1*-KI mice was similar to that observed in model mice for Huntington's disease and fALS. FoxO1 enhances autophagy by inducing the expression of autophagy-related genes in the nucleus and also by activating autophagy by associating with autophagy-related proteins in the cytoplasm (Cheng, 2019). In *Gfpt1*-KI mice, we observed that denervation increased FoxO1 expression at the mRNA level (Fig. 6E) but not at the protein level (Fig. 6F,G). Xbp1-s binds to FoxO1 and triggers proteasome-mediated degradation of FoxO1 in mouse embryonic fibroblasts (Zhou et al., 2011). Similarly, abnormally elevated Xbp1-u binds to active acetylated FoxO1, which is then degraded by the 26S proteasome in cancer cells (Zhao et al., 2013). In *Gfpt1*-KI mice, we showed that both Xbp1-u and Xbp1-s were increased in denervated GAS muscles in *Gfpt1*-KI mice (Fig. 6H,I), which were likely to have enhanced FoxO1 degradation (Fig. 6G). In addition, suppressed phosphorylation of Akt decreases phosphorylation of FoxO1 (p-FoxO1), and increases nuclear translocation and expression of FoxO1, which subsequently enhances autophagy (Li et al., 2020; Luo et al., 2023). Conversely, p-Akt-induced phosphorylation of FoxO1 causes the nuclear exclusion of FoxO1 and facilitates its proteasome-mediated degradation, which subsequently suppresses autophagy (Matsuzaki et al., 2003). The level of p-Akt was higher in denervated *Gfpt1*-KI mice compared to that in denervated WT mice (Fig. 6A,C), which suggested that FoxO1 was subjected to cytosolic degradation in *Gfpt1*-KI mice. Collectively, in denervated skeletal muscles in *Gfpt1*-KI mice, enhanced degradation of FoxO1 attenuated autophagy and increased misfolded protein aggregates, which made UPR maladaptive (Fig. 7).

Under the UPR, Xbp1-s induces the expression of Gfpt1, which increases the generation of UDP-GlcNAc and enhances proper protein glycosylation to suppress cell death (Wang et al., 2014). Indeed, in skeletal muscles in WT mice, denervation-mediated UPR increased *Gfpt1* expression and restrained Grp78-p62 colocalization (Fig. 4D,E,H). Thus, Gfpt1 is one of the key molecules to maintain protein homeostasis under UPR. In contrast, in *Gfpt1*-KI mice, Gfpt1 expression does not respond to UPR induction, which causes compromised global protein glycosylation and its homeostasis. Misfolded proteins cannot be removed efficiently, which leads to an endless loop of misfolded protein aggregation and phenotype worsening (Fig. 7).

Taken together, in both patients with *GFPT1*-CMS and aged *Gfpt1*-KI mice, lack of UDP-GlcNAc due to lack of Gfpt1 and lack of induction of Gfpt1 by the UPR are likely to compromise secure protein homeostasis and lead to maladaptive UPR in skeletal muscles.

## MATERIALS AND METHODS

### Generation of *Gfpt1*-KI mice

All mouse experiments were approved by the Animal Care and Use Committee of Nagoya University and were performed in accordance with relevant guidelines. C57BL/6J mice (Japan SLC) were introduced with the c.716dupG mutation in *Gfpt1* using the CRISPR/Cas9 technique. Briefly, the target sequence in the single-strand guide RNA (sgRNA, 5′-ATCCACATGGTGGGGATCACAGG-3′) was determined using the CRISPOR website (http://crispor.tefor.net/) by submitting the target sequence of the mouse *Gfpt1* gene (Chr6: 87,060,815-87,060,816, according to GRCm38/mm10). The single-stranded donor oligonucleotide (ssODN, 5′-CATCCTTAGCTAGGACTCAGATTGGATCCACATGGTGGGGGATCACAGGCAGAACGAGGTGGGAATGCACTCTGCACGGAT-3′) (FASMAC) was designed to include a target c.716dupG mutation. A mixture of 8 μM sgRNA and 200 ng/μl Cas9 proteins (New England Biolabs) was incubated at 37°C for 20 min to form a ribonucleoprotein complex, and the ssODN was added to the mixture to a final concentration of 250 ng/μl. Then, the mixture was electroporated into the cytoplasm of fertilized eggs using a NEPA21 electroporator (Nepa Gene). The injected eggs were then transferred into the oviductal ampulla of pseudo-pregnant ICR females (The Jackson Laboratory Japan). All manipulations for generating the mouse line were performed by the Animal Facility of Nagoya University Graduate School of Medicine following their general procedures. PCR amplification of the gene sequence around c.716 was performed with GoTaq enzyme (Promega) and the primers are listed in Table S1. Mutations in the *Gfpt1* gene in offspring were confirmed by Sanger sequencing. Potential off-target sites were predicted by the CRISPOR website. The top eight high-scored sites according to the off-target score were sequenced and no artifacts were detected in these sites (Table S2). Thus, a mouse line carrying the c.716dupG mutation in *Gfpt1* exon 9 was obtained. First, heterozygous males and heterozygous females were mated to breed mice homozygous for c.716dupG. We obtained 28 homozygous pups (27.2%) out of 103 pups, which indicated that the homozygosity did not affect the birth rate. We then mated homozygous males and homozygous females to obtain homozygous mice (*Gfpt1*-KI mice). The control mice were wild-type C57BL/6J mice purchased from Japan SLC. We observed that no *Gfpt1*-KI mice died before age 12 months, but lifespans were not quantitatively analyzed. Both male and female mice were used in analyzing the motor performances (Fig. 2A) and in counting the number of junctional folds by electron microscopy (Fig. 2L). In contrast, only male mice were used in the other analyses. Wild-type mouse pups were obtained by mating C57BL/6J mice, and were grown and aged in the same environment as that for *Gfpt1*-KI mice.

### Sciatic nerve transection

Mice were anesthetized with 3.5-4.0% isoflurane. The sciatic nerve at the right hindlimb was exposed and a 3-4 mm segment was excised. The severed nerve was tied with 5-0 black silk Ethilon suture (Ethicon) at both ends to prevent nerve reattachment. Sham surgery was performed in the left hindlimb without cutting the nerve. No analgesics were added after surgery. After the surgery, food was directly placed on the cage floor and a long tube was attached to the water bottle so that the operated mice could easily access the food and water.

### RNA extraction, reverse-transcription PCR and qRT-PCR

Skeletal muscle, heart and liver tissues were chopped into pieces and homogenized using a FastPrep 24 Instrument (MBP) and Lysing Matrix A tubes (MP Biomedical). Total RNA was extracted from homogenized tissues using TRIzol reagent (Themo Fisher Scientific) followed by the RNeasy Mini Kit (Qiagen), according to the manufacturer's directions. Total RNA was reverse transcribed into cDNA using random hexamers (Thermo Fisher Scientific) and ReverTra Ace reverse transcriptase (Toyobo) according to the manufacturer's instructions. PCR amplifications were performed by GoTaq (Promega). PCR products were run on a 2% agarose gel and visualized under ultraviolet light using the AE-9000 E-Graph Gel Documentation System (ATTO). qRT-PCR was performed using TB Green Premix ExTaq II (Takara Bio) on LightCycler 480 (Roche Diagnostics). The primer pairs used for reverse-transcription PCR (RT-PCR) and qRT-PCR are given in Table S1.

### High-throughput RNA-seq

Total RNA was extracted from the triceps brachii muscles, as stated above, at 12 months of age from WT and *Gftp1*-KI mice. RNA-seq was performed as previously described (Farshadyeganeh et al., 2023). Briefly, the quality of RNA was examined by an Agilent TapeStation, and the following thresholds were applied: quantity >50 ng; concentration >1 ng/ml; no contamination of DNA; and RNA integrity number >8.5. RNA-seq was performed at Macrogen, where a sequencing library was prepared using the TruSeq Stranded mRNA kit (Illumina). The library was read on an Illumina NovaSeq 6000 (150 bp paired-end reads). Raw reads were trimmed by Trimmomatic v0.39 (Bolger et al., 2014). Transcripts per million of each gene was calculated by Salmon v1.5.0 (Patro et al., 2017) with default parameters, and then, differential gene expression between WT and *Gfpt1*-KI mice was analyzed by DESeq2 v1.32.0 (Love et al., 2014). The RNA-seq data were deposited in the DDBJ Sequence Read Archive (DRA) with the BioProject accession numbers PRJDB16565 for WT mice and PRJDB18379 for *Gfpt1*-KI mice.

### Western blot analysis

Skeletal muscle tissues were chopped into pieces and homogenized in lysis buffer (50 mM KH_2_PO_4_, 10 mM EDTA, 5 mM reduced L-glutathione, 12 mM D-glucose-6-phosphate Na_2_, pH 7.6) with protease inhibitors (1 μM PMSF, 1 μg/ml leupeptin, 1 μg/ml pepstatin A), phosphatase inhibitor (PhosSTOP, Sigma-Aldrich) and *O*-GlcNAcase inhibitor (1 μM PUGNAc, Funakoshi) using a disposable homogenizer (BioMasher II, Funakoshi). The lysate was sonicated three times for 15 s using a UR-20P sonicator (Tomy Seiko). The supernatant containing protein lysate was collected by centrifugation at 19,000 ***g*** for 20 min at 4°C. Protein concentration was measured using the Pierce 660 nm Protein Assay Reagent (22660, Thermo Fisher Scientific) according to the manufacturer's recommendations. The protein concentrations were matched to 2.5 mg/ml across all muscle tissues. An equal volume of 2× SDS sample buffer [4% (w/v) SDS, 20% (v/v) glycerol, 0.01% (w/v) bromophenol blue and 0.125 M Tris-HCl (pH 6.8)] was added to each sample and denatured on a heat block at 95°C for 5 min.

The samples were loaded on a 7.5% SDS-polyacrylamide gel and separated by electrophoresis at 7 W for 1 h. Proteins were transferred onto a polyvinylidene difluoride (PVDF) membrane at 770 mA for 1.5 h at 4°C. The membranes were incubated in a blocking buffer (3% bovine serum albumin in 1× Tris-buffered saline with 0.1% Tween 20; TBS-T) for 1 h at room temperature (RT). The membranes were subsequently incubated with the following primary antibodies in TBS-T at 4°C overnight: rabbit monoclonal anti-GFPT1 antibody (1:1000, ab125069, Abcam), mouse monoclonal anti-RL2 antibody (1:800, sc-59624, Santa Cruz Biotechnology), mouse monoclonal anti-KDEL antibody (1:1000, sc-58774, Santa Cruz Biotechnology), mouse monoclonal anti-Chop antibody (1:2000, 2895, Cell Signaling Technology), rabbit monoclonal anti-phospho-eIF2α antibody (1:1000, 9721, Cell Signaling Technology), rabbit monoclonal anti-eIF2α antibody (1:1000, 5324, Cell Signaling Technology), rabbit monoclonal anti-Bax antibody (1:2000, 2772, Cell Signaling Technology), rabbit polyclonal anti-p62 antibody (1:1000, PM045, MBL), mouse monoclonal anti-Hsp70 antibody (1:1000, ADI-SPA-810-D, Enzo Life Sciences), rabbit monoclonal anti-LC3B antibody (1:1000, ab192890, Abcam), mouse monoclonal anti-MuRF1 antibody (1:1000, sc-398608, Santa Cruz Biotechnology), rabbit monoclonal anti-p-AMPKα (Thr172) antibody (1:1000, 2535, Cell Signaling Technology), rabbit monoclonal anti-AMPKα antibody (1:1000, 5831, Cell Signaling Technology), rabbit monoclonal anti-p-AKT (Ser473) antibody (1:1000, 4060, Cell Signaling Technology), rabbit monoclonal anti-AKT antibody (1:1000, 4691, Cell Signaling Technology), rabbit polyclonal anti-p-mTORC1 (Ser2448) antibody (1:1000, 2971, Cell Signaling Technology), rabbit polyclonal anti-mTOR antibody (1:1000, 2972, Cell Signaling Technology), rabbit monoclonal anti-FoxO1 antibody (1:1000, 2880, Cell Signaling Technology), rabbit monoclonal anti-Xbp1 antibody (1:1000, ab220783, Abcam) and rabbit polyclonal anti-Gapdh antibody (1:2000, G9545, Sigma-Aldrich). After draining the primary antibodies, the membranes were washed three times with TBS-T for 10 min at RT and incubated with the following secondary antibodies in TBS-T for 1 h at RT: horseradish peroxidase-conjugated goat anti-mouse IgG (1:2000, 7076, Cell Signaling Technology) and goat anti-rabbit IgG (1:2000, 7074, Cell Signaling Technology). The membranes were washed three times with TBS-T for 10 min at RT. Protein bands were detected with the ImageQunat LAS4000 Mini system (GE Healthcare Life Sciences). Uncropped blots are shown in Fig. S5.

### LC-MS/MS analysis of UDP-HexNAc and CMP-NeuAc

UDP-HexNAc in skeletal muscles was quantified as described before (Farshadyeganeh et al., 2023). We also quantified CMP-NeuAc in skeletal muscle (Nakajima et al., 2010). Briefly, weighed muscle tissues (20-40 mg) were quickly frozen in liquid nitrogen, and cell extracts for nucleotide sugar analysis were prepared. Hydrophilic interaction liquid chromatography and electrospray tandem mass spectrometry (HILIC-ESI-MS/MS) was performed on an LCMS-8060 (Shimadzu) coupled with a Nexera HPLC system (Shimadzu). Chromatography was performed on a BEH-amido column (2.1 mm internal diameter×150 mm, 3 mm; Waters) (Del Solar et al., 2020; Harada et al., 2021). Nucleotide sugars were analyzed in the multiple reaction monitoring mode with THE specific precursor ion [M-H]^−^ and product ions pairs as follows: m/z 606.1→384.7 for UDP-HexNAc; m/z 613→322 for CMP-NeuAc. The nucleotide sugar levels were indicated as pmol/mg tissue.

### Rotarod motor performance test

Muscle weakness and fatigability were measured using a rotarod machine (3-cm diameter rod, model 47600, Ugo Basile). Two days before the training session, the mice were habituated to the task. The training session consisted of three trials for each mouse separated by 10 min inter-trial intervals. The 10-min interval masked the fatigability of the mice, if any, and there was no shortening in the latency to fall in the three trials. The speed of the rod was linearly accelerated from 4 to 40 rpm in 4 min. The average of the latency to fall in three trials was recorded.

### Immunofluorescence staining

Mice were initially exposed to 3.5-4.0% isoflurane, and the blood was exchanged with 4% paraformaldehyde (PFA) under 2.0-3.0% isoflurane. Mice were euthanized by this exsanguination procedure. Muscle tissues were dissected and fixed in 4% PFA at 4°C overnight. PFA was substituted for 15% sucrose and then 30% sucrose for more than 4 h each successively for dehydration. Tissues were put in isopentane in liquid nitrogen for 30 to 45 s. Frozen tissues were cut into 10 µm-thick sections using a cryomicrotome (CM3050S, Leica Microsystems). Tissue sections were kept in Milli-Q water at 4°C.

Tissue sections were washed with phosphate-buffered saline (PBS) three times for 5 min and blocked in 5% goat serum for 30 min at RT. After blocking, the tissue sections were incubated with the following primary antibodies in 5% goat serum at 4°C overnight: rat monoclonal anti-laminin α2 antibody (1:50, sc-59854, Santa Cruz Biotechnology), mouse monoclonal anti-KDEL antibody (1:200, sc-58774, Santa Cruz Biotechnology) and rabbit polyclonal anti-p62 antibody (1:200, PM045, MBL). On the following day, the sections were washed with PBS five times for 5 min at RT and incubated with the following secondary antibodies for 2 h at RT: Alexa Fluor 594-conjugated goat anti-rat IgG (1:100, A11007, Thermo Fisher Scientific), Alexa Fluor 594-conjugated goat anti-mouse IgG (1:100, A11005, Thermo Fisher Scientific) and Alexa Fluor 488-conjugated goat anti-rabbit IgG (1:1000, A11034, Thermo Fisher Scientific). The sections were washed with PBS five times for 5 min at RT and mounted with VECTASHIELD Antifade Mounting Medium with DAPI (H-1200, VectorLabs).

### Visualization and quantification of AChR clusters

The GAS and triceps brachii muscles were fixed in ice-cold 4% PFA overnight and teased into fibers in PBS. The teased fiber was incubated in 100 mM glycine in PBS for 15 min at RT. The fiber was washed in PBS three times for 10 min and blocked in 2% bovine serum albumin, 5% goat serum and 0.5% Triton X-100 in PBS for 1 h at RT. After blocking, the fiber was incubated with rabbit monoclonal anti-synaptophysin antibody (1:50, 18-0130, Thermo Fisher Scientific) at 4°C overnight. On the following day, the fiber was washed with 0.1% Triton X-100 in PBS (PBS-X) three times for 30 min at RT and incubated with Alexa Fluor 488-conjugated goat anti-rabbit IgG (1:1000, A11034, Thermo Fisher Scientific) and Alexa Fluor 594-conjugated α-bungarotoxin (1:1000, B13423, Thermo Fisher Scientific) for 1 h at RT. The fiber was washed with PBS-X three times for 1 h at RT. Lastly, the fiber was placed on a glass slide and mounted with VECTASHIELD Antifade Mounting Medium with DAPI (H-1200, VectorLabs). The NMJ was visualized under the BX53-34-FL-3 system microscope (Olympus) and was quantified by two masked researchers using MetaMorph software (Molecular Devices).

### Electron microscopy

The triceps brachii muscles were fixed in 4% PFA overnight at 4°C. To confirm the presence of the NMJ, acetylcholinesterase staining using the Ellman method was performed. Briefly, whole muscle was incubated with 5 mg/ml acetylthiocholine iodide in a buffer containing 0.82% sodium acetate, 0.6% acetic acid, 2.94% sodium citrate, 0.75% copper sulphate and 0.165% potassium ferricyanide at 37°C for 2-4 h until the NMJ was stained. After washing with Milli-Q water three times, the stained muscles were cut into ∼1 mm^2^ pieces where the NMJs were supposed to be present, and the excised blocks were fixed with 2% glutaraldehyde for 2 h, treated with 1% OsO_4_, dehydrated in ethanol and embedded in Epon 812 (TAAB). Ultrathin (60-70 nm) sections were collected from blocks and stained with uranyl acetate and lead citrate. The NMJ was identified by inspecting the entire ultrathin sections using a JEM-1400PLUS Transmission Electron Microscope.

### C2C12 cell culture and transfection

C2C12 myoblasts (RCB0987) were freshly purchased from RIKEN BioResource Center Cell Bank. C2C12 myoblasts were seeded in collagen I-coated dishes and grown in Dulbecco's modified Eagle medium (DMEM; Thermo Fisher Scientific) supplemented with 10% fetal bovine serum (Thermo Fisher Scientific) at 37°C with 5% CO_2_. At ∼80% confluency, myoblasts were differentiated into myotubes in DMEM supplemented with 2% horse serum (Thermo Fisher Scientific) for 6 days. On the fourth day of differentiation, 50 μM siRNA duplexes were introduced by Lipofectamine RNAiMAX transfection reagent (Thermo Fisher Scientific) according to the manufacturer's directions.

### CCK-8 assay to quantify viable cells

CCK-8 assay (Dojindo) was performed according to the manufacturer's directions to estimate cell viability. Briefly, C2C12 myoblasts in 100 μl DMEM supplemented with 10% fetal bovine serum were seeded in a 96-well plate. After incubation for 48 h at 37°C, 10 μl CCK-8 solution (Dojindo) was added to the cells and they were incubated at 37°C for 1 h. Absorbances were measured at 450 nm using the BioTek Cytation 5 Cell Imaging Multimode Reader (Agilent).

### Statistical analysis

Western blots, the cross-section areas of myofibers and the areas of AChR cluster were quantitatively analyzed using ImageJ (Laboratory for Optical and Computational Instrumentation, University of Wisconsin-Madison). Statistical analyses were performed with GraphPad Prism 9.5.1 (GraphPad Software). Statistical significance was calculated by unpaired two-tailed Student's *t*-test, multiple unpaired two-tailed *t*-test, one-way ANOVA followed by Tukey's post hoc test or two-way ANOVA followed by Sidak's post hoc test. *P*-values of 0.05 or less were considered statistically significant.

## Supplementary Material

10.1242/dmm.050768_sup1 Supplementary information
