# Supplementary material for: Muscle-specific lack of Gfpt1 triggers ER stress to alleviate misfolded protein accumulation
Source: Dis Model Mech. 2024 Jul 25;17(8):dmm050768. doi: 10.1242/dmm.050768 (PMC11554261; doi:10.1242/dmm.050768)
Supplement: Supplementary information [file dmm-17-050768-s1.pdf]

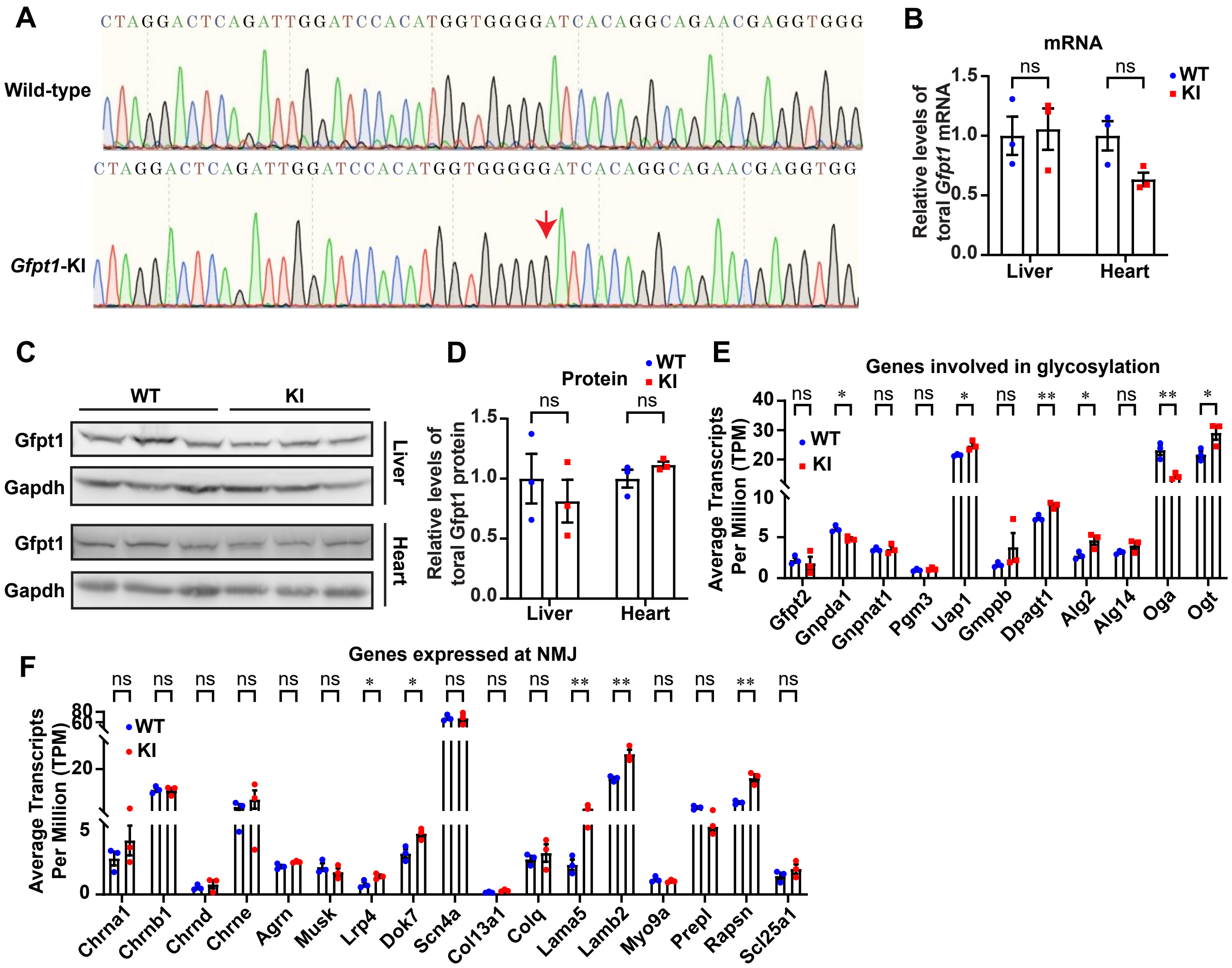


**Fig. S1. Sequencing chromatograms and gene expression profiles in WT and *Gfpt1*-KI mice**

**(A)** Sequencing chromatograms showing the insertion of a nucleotide G in homozygous *Gfpt1*-KI mouse. Arrows point to the insertion of nucleotide G. **(B)** Quantitative RT-PCR of total *Gfpt1* in the liver and the heart in WT and *Gfpt1*-KI mice ($n$ = 3 mice each). **(C, D)** Representative immunoblots and quantification of Gfpt1 protein in the liver and the heart in WT and *Gfpt1*-KI mice ($n$ = 3 mice each). **(E, F)** RNA-seq analysis of triceps brachii in WT and *Gfpt1*-KI mice at 12 months of age ($n$ = 3 mice each). Eleven representative genes involved in glycosylation and seventeen representative genes at the NMJ are indicated. **(B, D, E, F)** Mean and SEM are indicated. *$p < 0.05$, **$p < 0.01$, ***$p < 0.001$, and ****$p < 0.0001$ by two-way ANOVA followed by Sidak's posthoc test.

Disease Models & Mechanisms • Supplementary information

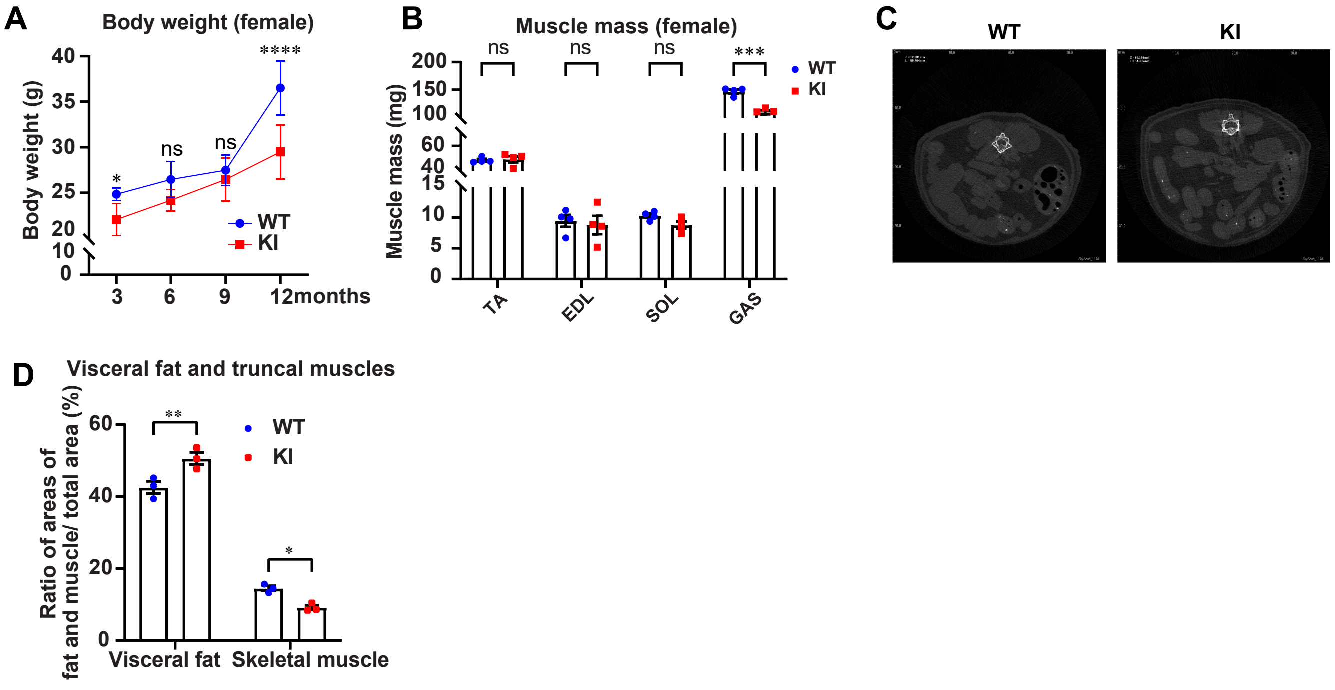


**Fig. S2. Body weights, muscle masses, and truncal CT images in WT and *Gfpt1*-KI mice**

**(A)** Temporal profile of body weights in female WT and *Gfpt1*-KI mice at indicated ages ($n$ = 6 to 13 mice each). *$p < 0.05$ and ****$p < 0.0001$ by two-way repeated measures ANOVA followed by Sidak's posthoc test. Body weights of male mice are indicated in Figure 2B. **(B)** Weights of tibialis anterior (TA), extensor digitorum longus (EDL), soleus (SOL), and gastrocnemius (GAS) muscles in female WT and *Gfpt1*-KI mice at 12 months of age ($n$ = 4 mice). Muscle weights of male mice are indicated in Figure 2C. **(C, D)** Representative truncal CT images and quantification in WT and *Gfpt1*-KI mice at 12 months of age ($n$ = 3 mice each). **(A, B, D)** Mean and SEM are indicated. *$p < 0.05$, **$p < 0.01$, ***$p < 0.001$ and ****$p < 0.0001$ by two-way ANOVA followed by Sidak's posthoc test.

Disease Models & Mechanisms • Supplementary information

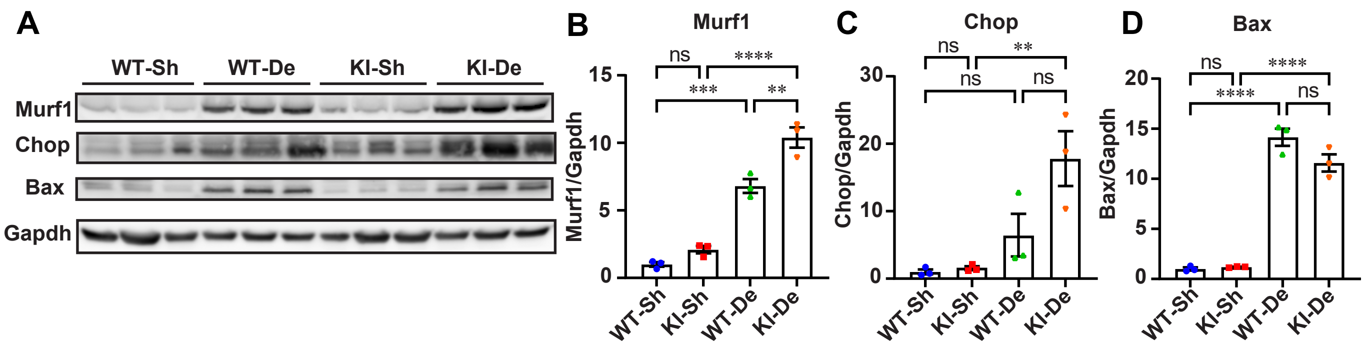


**Fig. S3. Denervation accelerated both ubiquitin-proteasome degradation and apoptosis in both WT and *Gfpt1*-KI mice**

**(A)** Representative immunoblots and quantification of Murf1 **(B)**, Chop **(C)**, and Bax **(D)** in the GAS muscles in WT and *Gfpt1*-KI mice (n = 3 mice each). Mean and SEM are indicated. **p* < 0.05, ***p* < 0.01, ****p* < 0.001, and *****p* < 0.0001 by one-way ANOVA followed by Tukey's posthoc test.

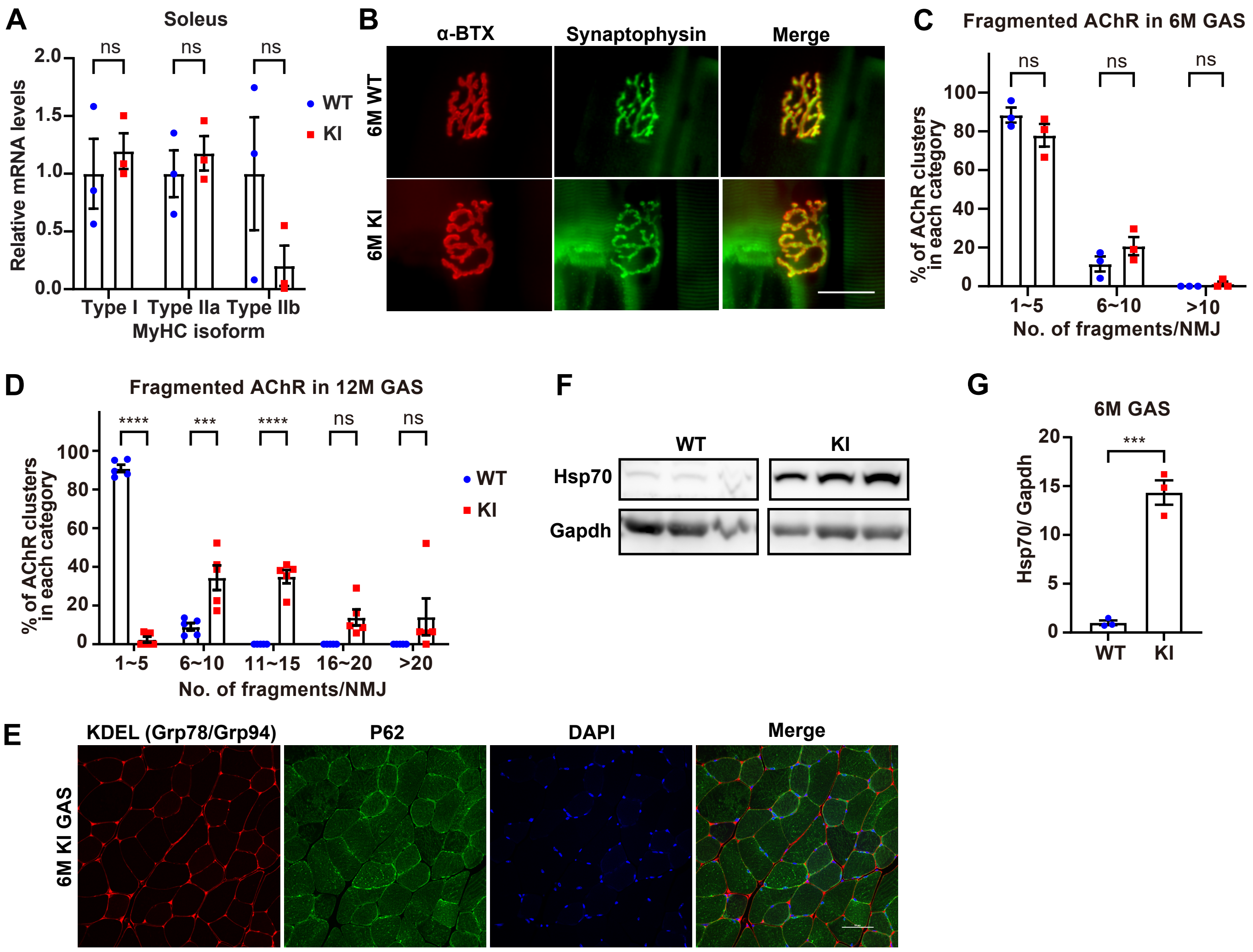


**Fig. S4. *Gfpt1*-KI mice at 6 months of age showed no NMJ abnormality or Grp78-p62 colocalization**

**(A)** Quantitative RT-PCR of *Myh7* (type I myofiber), *Myh2* (type IIA myofiber), and *Myh4* (type IIb myofiber) in soleus muscles in WT and *Gfpt1*-KI mice at 6 months of age ($n$ = 3 mice each). Mean and SEM are indicated. no significance by two-way ANOVA followed by Sidak's posthoc test. **(B)** Representative images of the NMJs stained with α-bungarotoxin (red) to label acetylcholine receptor (AChR) and synaptophysin (green) to label the nerve terminal in GAS muscles of mice in WT and *Gfpt1*-KI mice at 6 months of age. Bar = 50 µm. **(C, D)** The percentage of AChR clusters with the indicated numbers of fragments in GAS muscles in WT and *Gfpt1*-KI mice at 6 months of age ($n$ = 20 to 30 AChR clusters per mouse × 3 mice each) **(C)** and at 12 months of age ($n$ = 20 to 30 AChR clusters per mouse × 5 mice each) **(D)**. **(E)** Representative confocal images of the cross sectioned myofibers stained with antibodies against KDEL (Grp78/Grp94) and p62 in the GAS muscles in WT and *Gfpt1*-KI mice at 6 months of age. Bar = 50 µm. **(F, G)** Immunoblots and quantification of Hsp70 in the GAS muscles in WT and *Gfpt1*-KI mice at 6 months of age ($n$ = 3 mice each). Mean and SEM are indicated. ***$p < 0.001$ by unpaired t-test.

Disease Models & Mechanisms • Supplementary information

## Table S1. PCR primers and siRNA sequences

| Gene | Forward | Reverse | Annealing temperature | Size (bp) |
|---|---|---|---|---|
| **PCR primers** | | | | |
| *Gfpt1*-genotyping | AGCCCTCTCTTGATTGGTGT | TGCCAAAACATTTCCCCATAAAGT | 56˚C | 427 |
| *Gfpt1*-E9 | AGCCCTCTCTTGATTGGTGT | CAACTGCCTTTTCCTCAACAG | 56˚C | 154<br>202 |
| *Gfpt1*-T | AATGCTGGTCCTGAGATTGGCG | CCGAGCATGATCTCTTTGCGTC | 65˚C | 134 |
| *Hspa5* | TGCAGCAGGACATCAAGTTC | TACGCCTCAGCAGTCTCCTT | 57˚C | 157 |
| *Ddit3* | CCTAGCTTGGCTGACAGAGG | CTGCTCCTTCTCCTTCATGC | 57˚C | 196 |
| *Xbp1s* | CTGAGTCCGCAGCAGGTG | GACCTCTGGGAGTTCCTCCA | 58˚C | 188 |
| *Foxo1* | ACATTTCGTCCTCGAACCAGCTCA | ATTTCAGACAGACTGGGCAGCGTA | 64˚C | 161 |
| *Bax* | AAGCTGAGCGAGTGTCTCCGGCG | GCCACAAAGATGGTCACTGTCTGCC | 68˚C | 362 |
| *Myh7* | CTTCTACAGGCCTGGGCTTAC | CTCCTTCTCAGACTTCCGCAG | 58˚C | 128 |
| *Myh2* | TTCCAGAAGCCTAAGGTGGTC | GCCAGCCAGTGATGTTGTAAT | 57˚C | 94 |
| *Myh4* | CTTGTCTGACTCAAGCCTGCC | TCGCTCCTTTTCAGACTTCCG | 60˚C | 158 |
| *Gapdh* | GTTGTCTCCTGCGACTTCA | TGCTGTAGCCGTATTCATTG | 55˚C | 124 |
| **siRNAs** | | | | |
| siControl | UUCUCCGAACGUGUCACGUtt | ACGUGACACGUUCGGAGAAtt | - | - |
| siGfpt1 | GGAGAGAGUUAUCCAACAAtt | UUGUUGGAUAACUCUCUCCtt | - | - |

Disease Models & Mechanisms • Supplementary information

## Table S2. Top eight high-scored off-target sites by the CRISPOR website

| Off-target sequence | Chr | start | end | Forward primer | Reverse primer | Annealing temp. (˚C) | Size (bp) |
|---|---|---|---|---|---|---|---|
| CATCACAGGGTGGGGATCACTGG | 9 | 45105637 | 45105659 | TGACAGGAGCAAACACAAGCCC | GAGACTCAGAGAAGAAAAGCACCCAG | 62 | 206 |
| ATCCCAATAGTGGGGATCACAGG | 11 | 96056967 | 96056989 | GGGGCTAGGGTCCAAACATGG | AGAAGAAAACCTCTATCTCCGCACCTG | 63 | 369 |
| ATCTTCTTGATGGGGATCACAGG | 18 | 9354314 | 9354336 | TGCTAACAGAGTAGAACACTGGGTCA | ACAATCATAAGGGCACCTGCTG | 60 | 341 |
| AACCCCAAGGAGGGGATCACTGG | 1 | 42687861 | 42687883 | CCACCACTAAACTACTTGATGGAAGTCT | AATAAGGCAAACACAGTCCTCAGC | 59 | 273 |
| CTCTCCATGGTAGGGATCACAGG | 13 | 49306493 | 49306515 | AGTCGCCCTTTTGCTATGCCA | ACGATGGACTGAGGGCAAACTG | 62 | 598 |
| TTCCACATGGTGGGGATTACAGG | 7 | 48831814 | 48831836 | AGGGGGCTGCTTCAATAGGAATG | AGAAAAGGAGAGAGTATGTGGGGTG | 61 | 368 |
| AACCTCCTGGTGGGGATCACAGG | 8 | 11116343 | 11116365 | GCTTGACTTGGAGAGCCATTCC | AAGCAAACAAGCCCCAGGTCAC | 62 | 698 |
| ATCATCATGGTGGGGAGCACAGG | 14 | 65075148 | 65075170 | CTTACTGGAAGCAGAAAGCCAATG | TTAGGAGTGAAGCCTTAGTGGAGG | 59 | 367 |

Disease Models & Mechanisms • Supplementary information

## WB-Raw blots

### Figure 1C

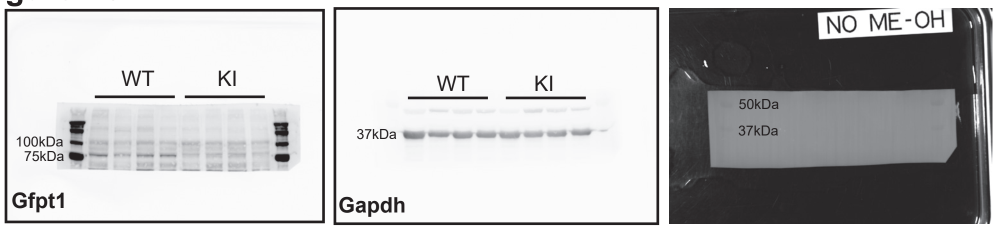


### Figure 1F

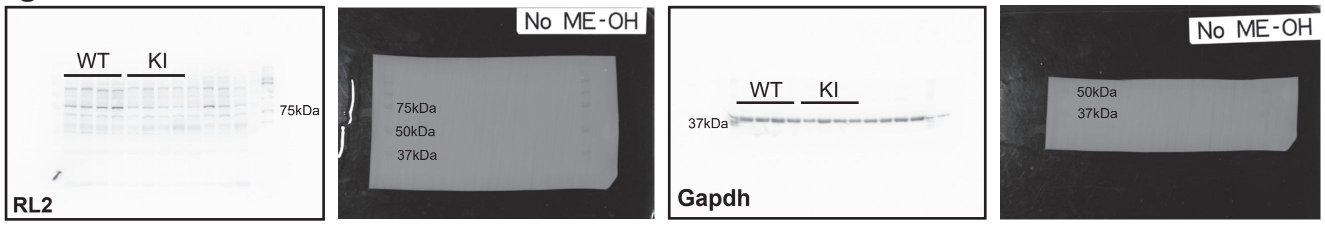


### Figure 3B

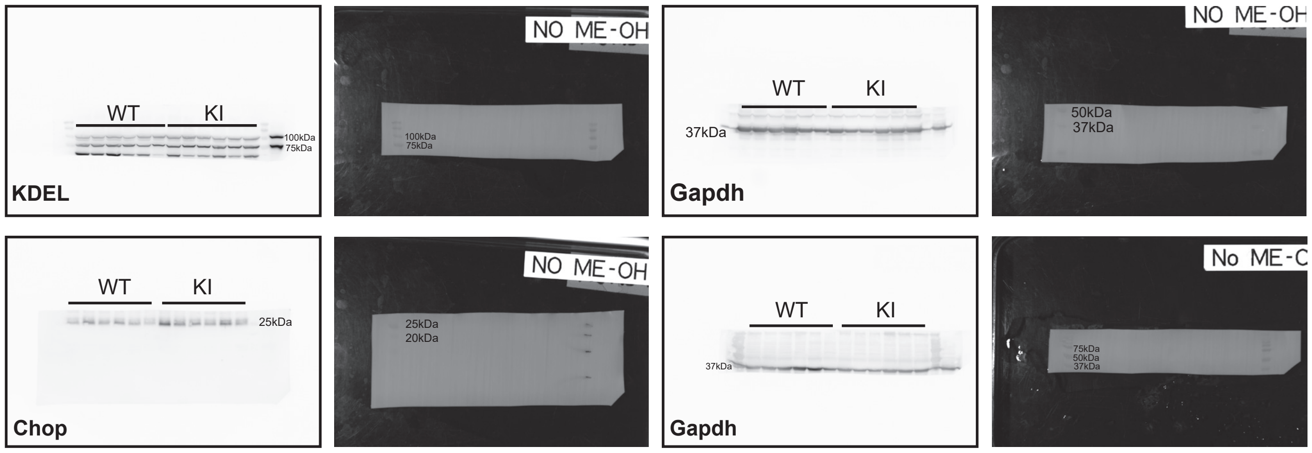


### Figure 3D

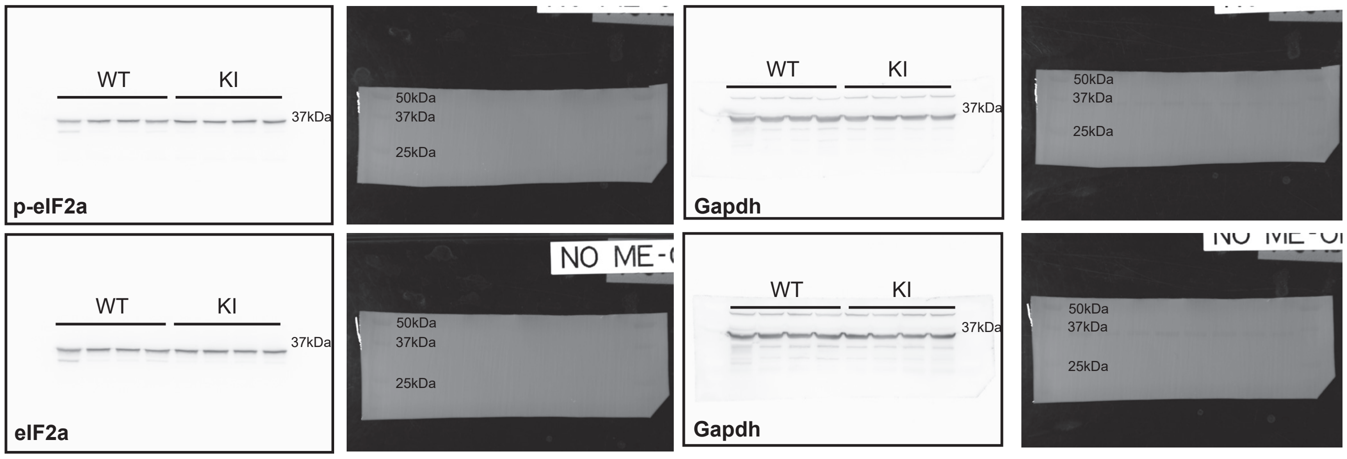


Disease Models & Mechanisms • Supplementary information

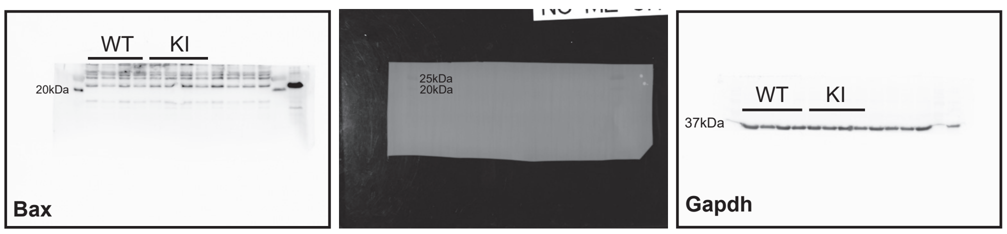


## Figure 4A

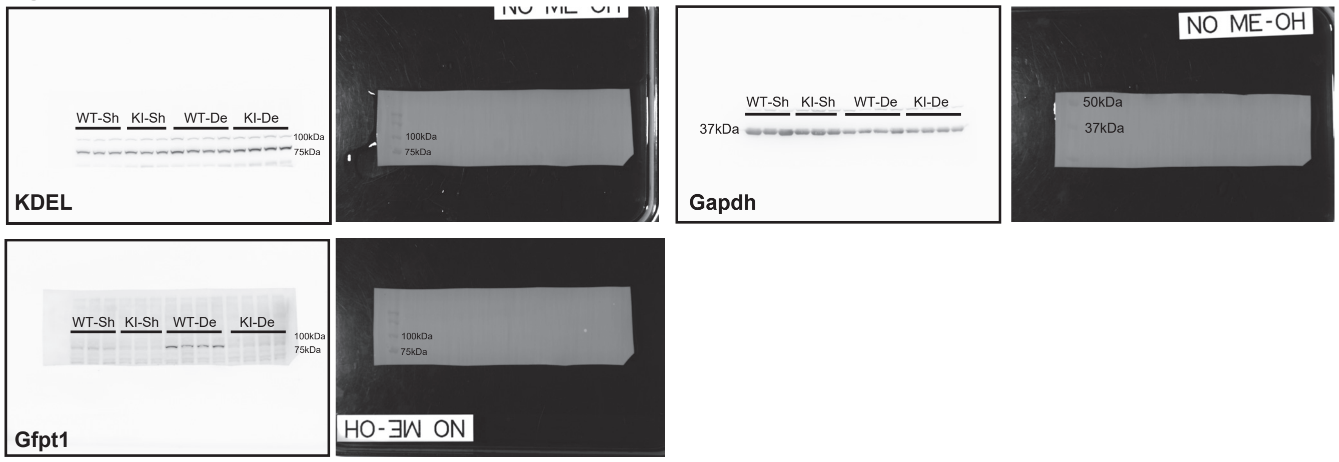


## Figure 4F

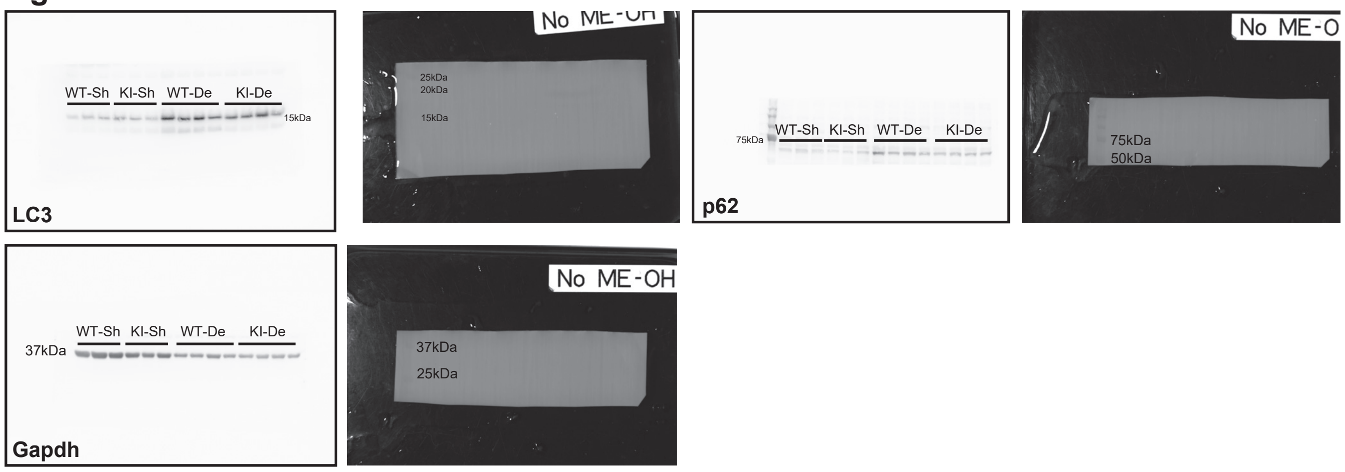


## Figure 4I

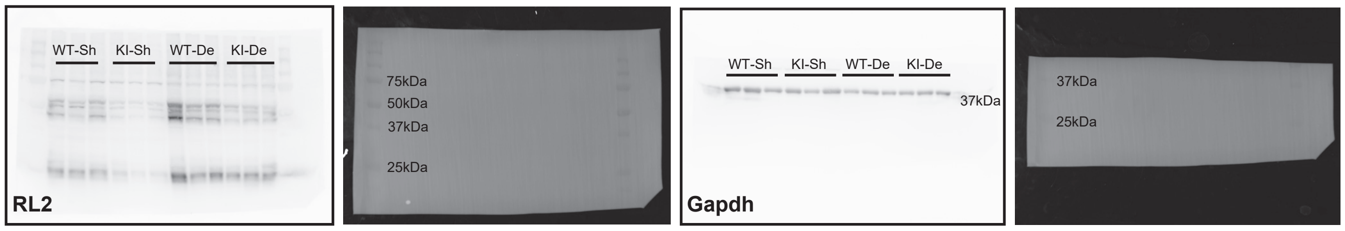


Disease Models & Mechanisms • Supplementary information

## Figure 5E

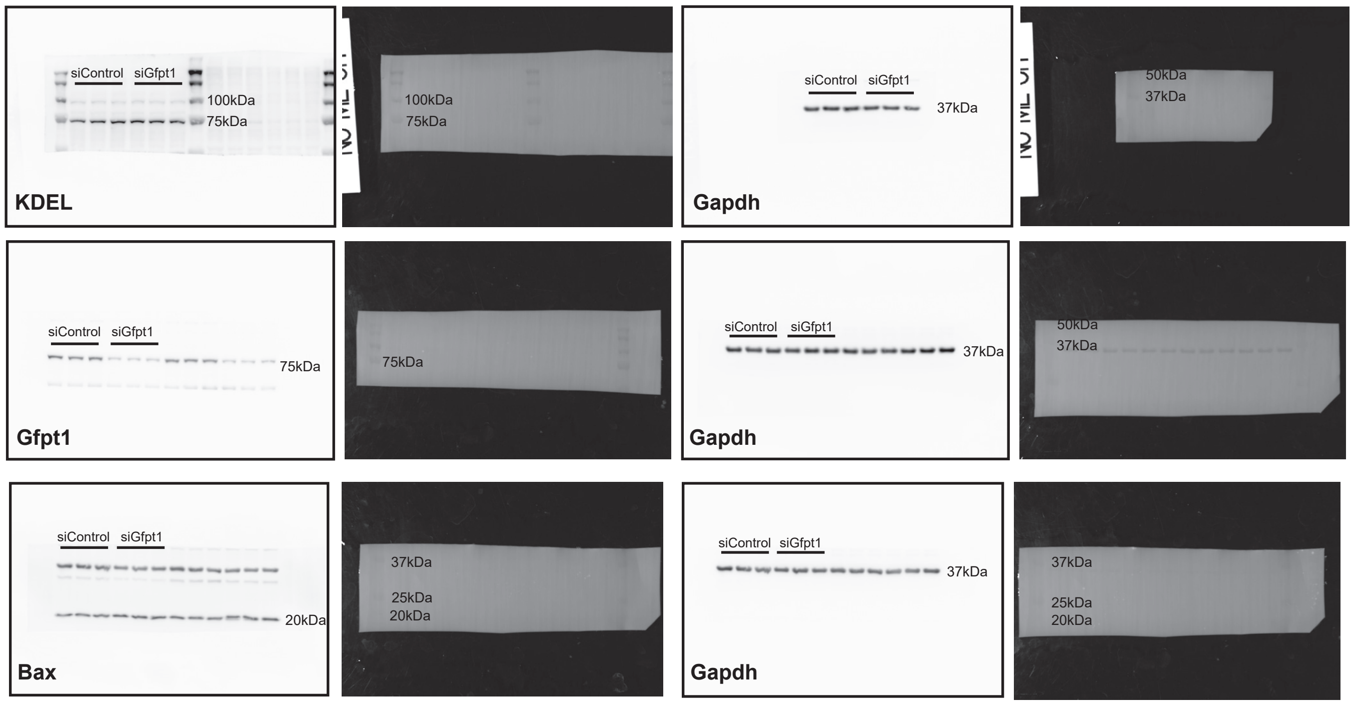


## Figure 5H

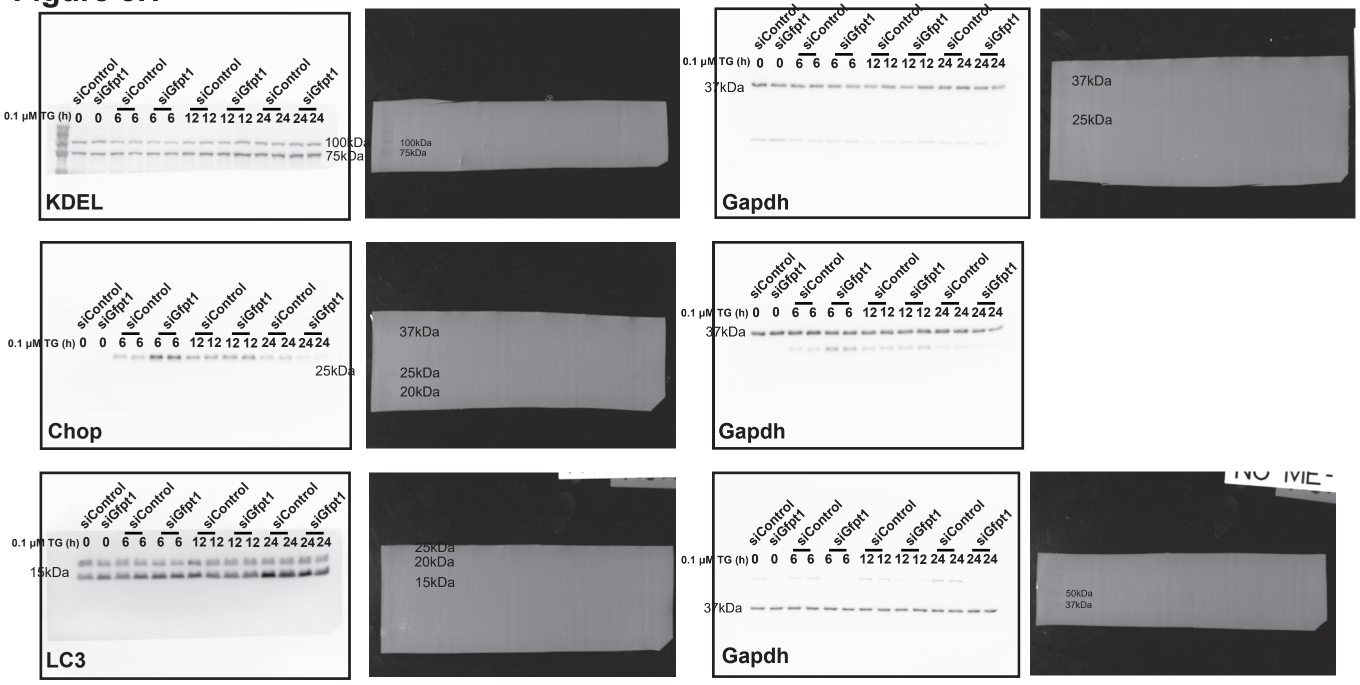


Disease Models & Mechanisms • Supplementary information

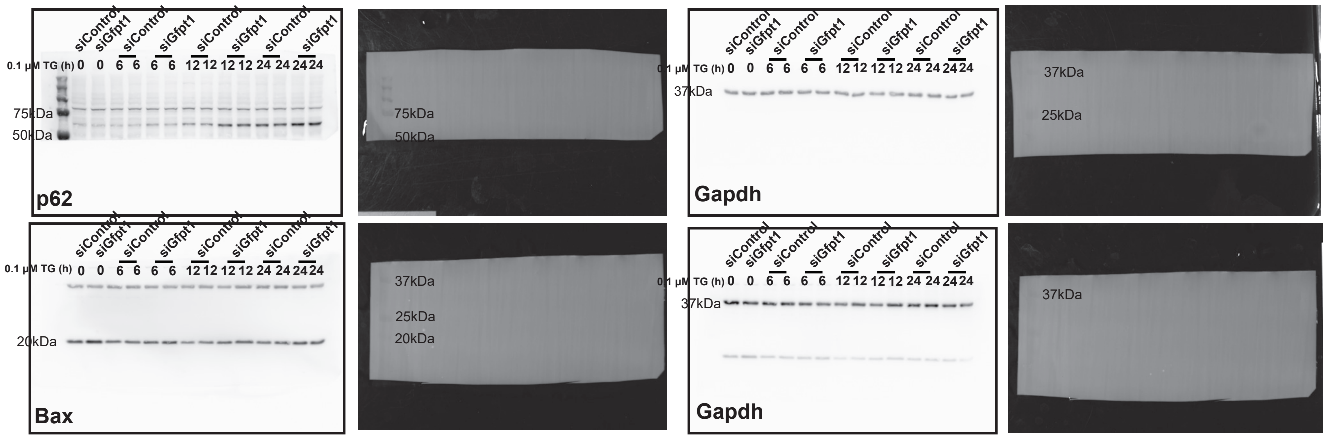


## Figure 6A

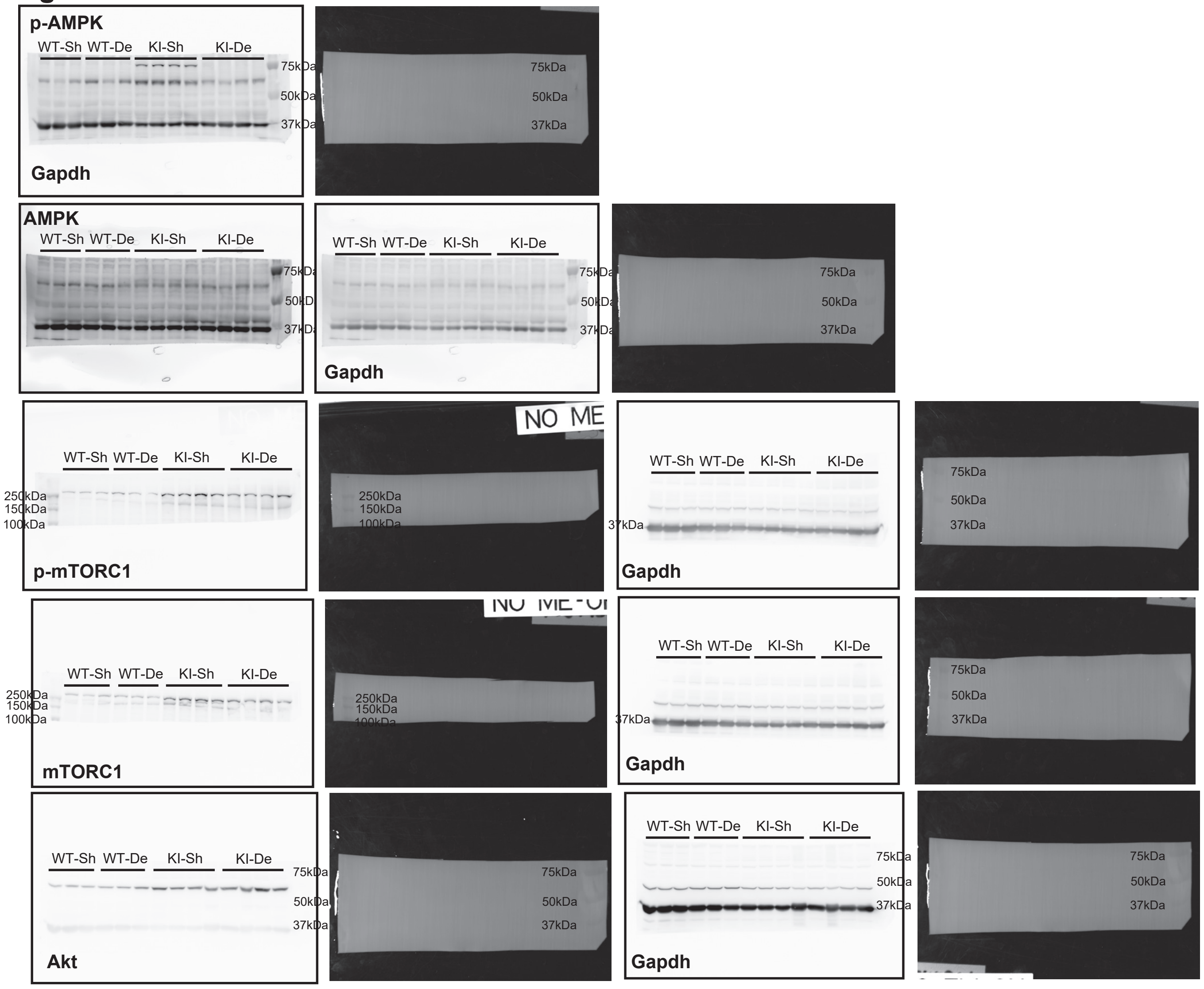


Disease Models & Mechanisms • Supplementary information

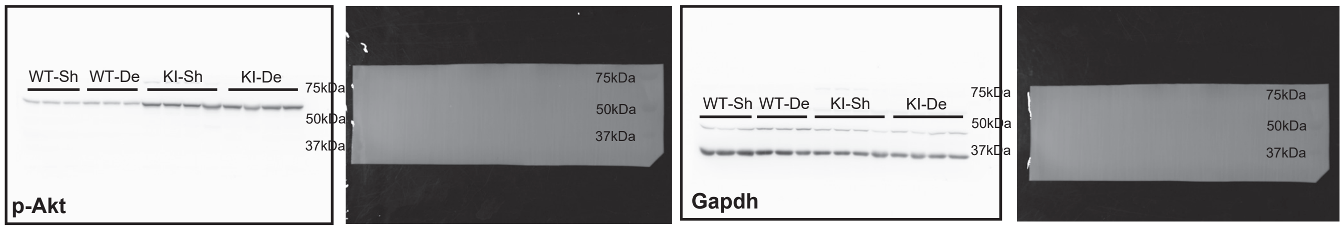


## Figure 6F

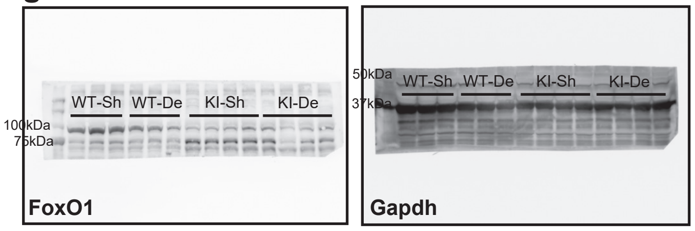


## Figure 6H

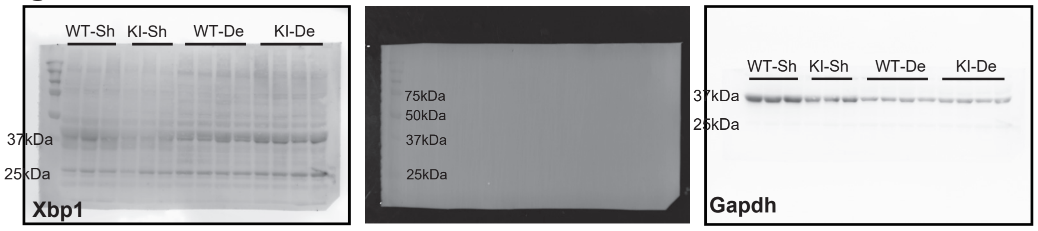


## Old version
## Figure 3D

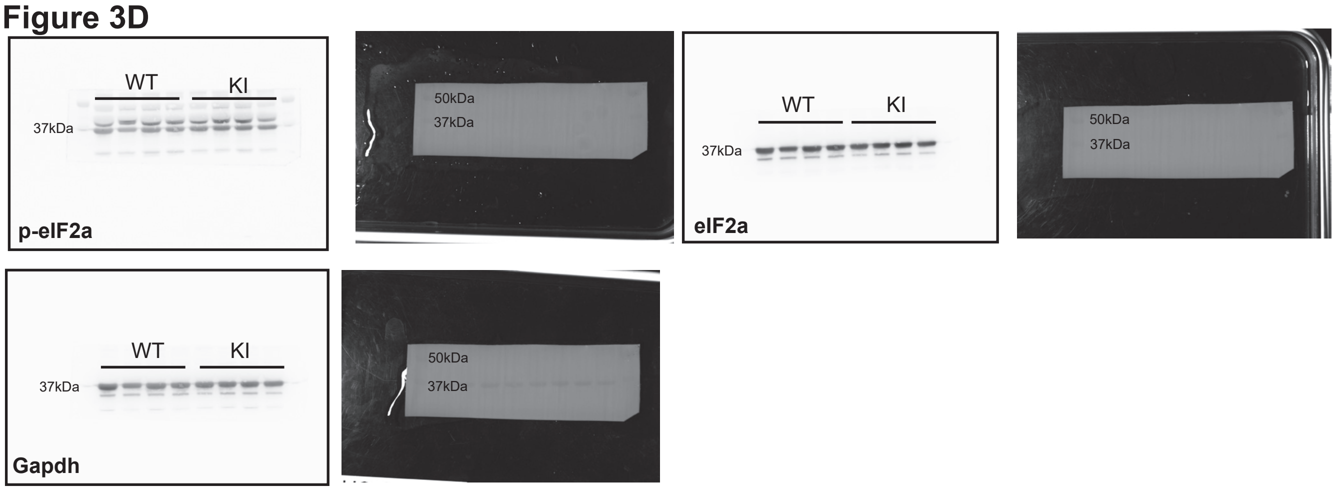


## Figure 6A

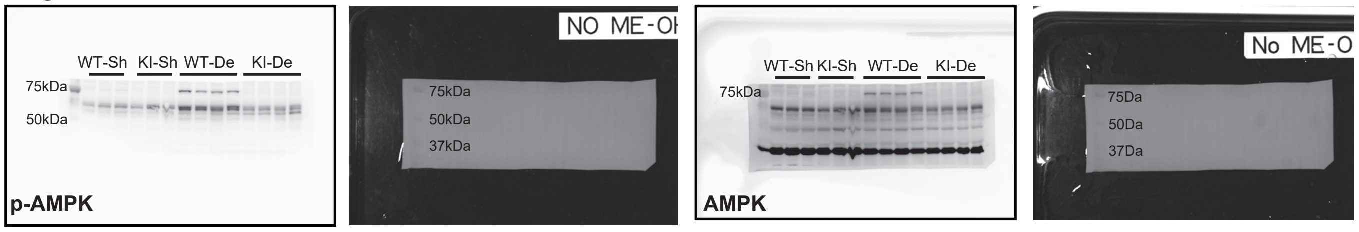


Disease Models & Mechanisms • Supplementary information

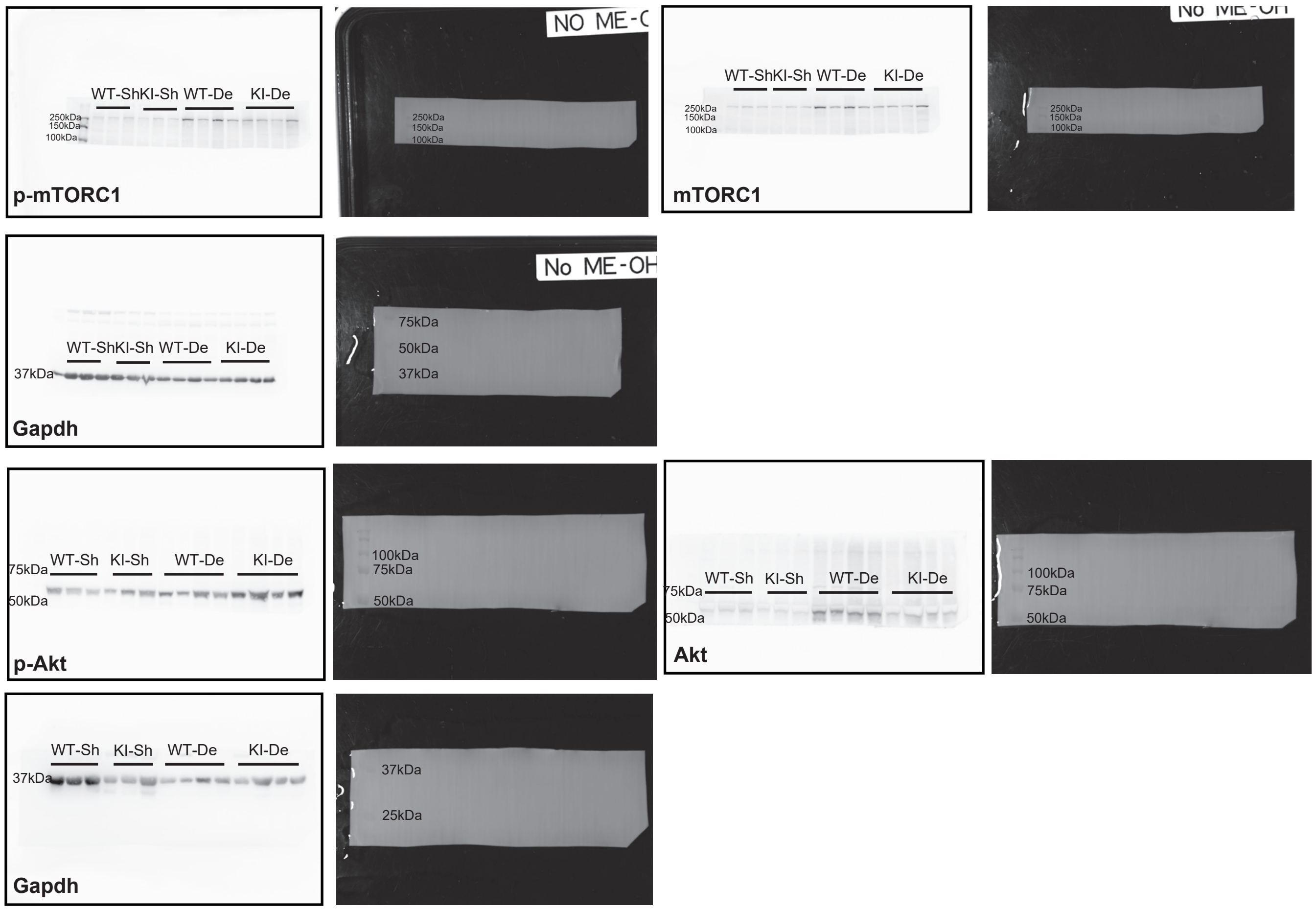


## Figure 6F

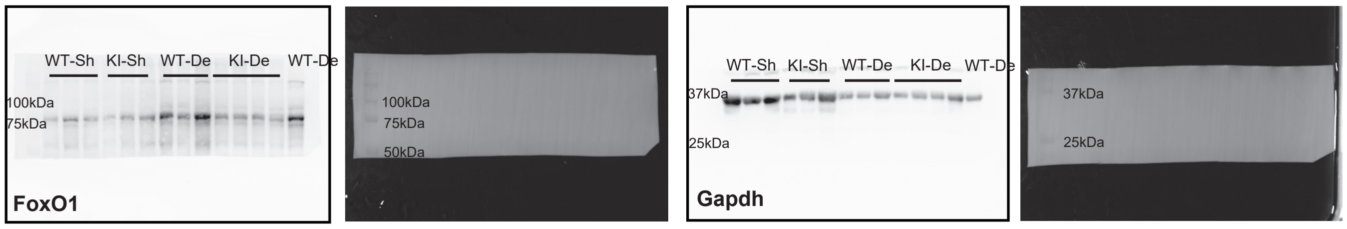


**Fig. S5. Blot Transparency**

Disease Models & Mechanisms • Supplementary information
